# *Hericium erinaceus* potentially rescues behavioural motor deficits through ERK-CREB-PSD95 neuroprotective mechanisms in rat model of 3-acetylpyridine-induced cerebellar ataxia

**DOI:** 10.1038/s41598-020-71966-z

**Published:** 2020-09-10

**Authors:** Pit Shan Chong, Sharafuddin Khairuddin, Anna Chung Kwan Tse, Lih Fhung Hiew, Chun Lok Lau, George Lim Tipoe, Man-Lung Fung, Kah Hui Wong, Lee Wei Lim

**Affiliations:** 1 Neuromodulation Laboratory, School of Biomedical Sciences, Li Ka Shing Faculty of Medicine, The University of Hong Kong, 21 Sassoon Road, Pokfulam, Hong Kong Special Administrative Region, China; 2 Department of Anatomy, Faculty of Medicine, University of Malaya, 50603 Kuala Lumpur, Malaysia

**Keywords:** Neuroscience, Diseases of the nervous system, Neurodegeneration, Translational research

## Abstract

Cerebellar ataxia is a neurodegenerative disorder with no definitive treatment. Although several studies have demonstrated the neuroprotective effects of *Hericium erinaceus* (H.E.), its mechanisms in cerebellar ataxia remain largely unknown. Here, we investigated the neuroprotective effects of H.E. treatment in an animal model of 3-acetylpyridine (3-AP)-induced cerebellar ataxia. Animals administered 3-AP injection exhibited remarkable impairments in motor coordination and balance. There were no significant effects of 25 mg/kg H.E. on the 3-AP treatment group compared to the 3-AP saline group. Interestingly, there was also no significant difference in the 3-AP treatment group compared to the non-3-AP control, indicating a potential rescue of motor deficits. Our results revealed that 25 mg/kg H.E. normalised the neuroplasticity-related gene expression to the level of non-3-AP control. These findings were further supported by increased protein expressions of pERK1/2-pCREB-PSD95 as well as neuroprotective effects on cerebellar Purkinje cells in the 3-AP treatment group compared to the 3-AP saline group. In conclusion, our findings suggest that H.E. potentially rescued behavioural motor deficits through the neuroprotective mechanisms of ERK-CREB-PSD95 in an animal model of 3-AP-induced cerebellar ataxia.

## Introduction

Cerebellar ataxia is a progressive neurodegenerative disorder that is characterised by degeneration of the cerebellum, leading to impaired balance, motor dysfunction, and limb and gait ataxia^1,2^. This disorder involves the altered organisation of cerebellar circuits and connectivity, and is often genetically inherited^3^. Previous studies have demonstrated that dysfunction and degeneration of Purkinje cells in the cerebellum largely contribute to cerebellar ataxia^4,5^. The Purkinje cell dysfunction, which involves the deficiency of Ca^2+^-activated K^+^ channels, has been shown to play a major role in locomotor abnormalities and disruption of motor coordination in an animal model of cerebellar ataxia^6^. The inability of Purkinje cell firing serves as a potential target for improving motor dysfunction in ataxic subjects. Although limited complete treatments are available for some rare forms of ataxia with well-studied biochemical defects^1^, only rehabilitative treatment is available for the majority of cases of ataxia. There is scant evidence to support pharmacological management as an effective treatment to improve cerebellar ataxia^7^. Persistent training with intensive exercises focusing on balance and locomotion can slow down the deterioration of balance and impairment of gait in ataxic patients^8^.

Antioxidants have been thoroughly tested for their efficacy to slow down the progressive deterioration in neurodegenerative disorders. Medicinal mushrooms comprise of an efficient antioxidant machinery due to their bioactive compounds such as polyphenols, alkaloids, terpenes, polysaccharides, carotenoids and various enzymes^9^. Functional foods such as culinary and medicinal mushrooms have been suggested to improve cerebellar ataxia^10^. In a clinical trial, four siblings aged between 34 to 50 with diagnosis of hereditary cerebellar ataxia were intramuscularly injected with mycelial extract of *Ganoderma capense,* which was shown to have significant effects on their functional ability^10^. *Hericium erinaceus* (Bull.: Fr.) Pers. (*H. erinaceus* or H.E.)*,* also commonly known as lion’s mane mushroom, monkey’s head mushroom, or Yamabushitake, has been used as a traditional treatments in China and Japan for thousands of years^11^. *Hericium erinaceus* possesses numerous beneficial effects including anti-cancer, immunomodulatory, antihyperglycemic, anti-hypercholesterolemic, neuroprotective, antimicrobial, anti-neuropathic, antidepressant, antioxidant, and anti-aging activities^12–15^. It has been widely investigated in *in-vitro* studies as a neurite outgrowth stimulator in neural hybrid clone NG108-15 and rat pheochromocytoma PC12^16–18^. Furthermore, *H*. *erinaceus* was found to promote the recovery of motor and sensory functions after crush injury, and enhanced the regeneration of peripheral nerves^19^. Additionally, *Hericium erinaceus* has also been shown to improve the hippocampal-dependent recognition memory with enhancement of neurotransmission and neurogenesis in the hippocampus and the cerebellum^13,20^. Recent *in-vivo* and clinical studies in early cognitive decline and depression suggest it has the potential to ameliorate cognitive and neurological deficits^21–26^.

Various bioactive components of *H*. *erinaceus* have been found to induce the release of neurotrophic factors including brain-derived neurotrophic factor (BDNF), pro-BDNF and nerve growth factors (NGF)^15,17,27–33^, which are neurotrophic factors involved in the regulation of neuronal survival, growth, and function. Besides their involvement in neuroplasticity, neurotrophic compounds can also stimulate neurogenesis to rescue neuronal deficits even in adulthood, as well as protect neurons from apoptosis^34,35^. Thus, *H. erinaceus* could be a suitable candidate for the treatment of cerebellar ataxia as it could potentially protect Purkinje cells from degeneration, as well as promote neuronal survival and growth. More importantly, toxicity study of subchronic oral doses of *H. erinaceus* for 90 days demonstrated no negative effects on behaviour, no adverse clinical signs, or mortality, indicating it could be a potentially safe treatment for cerebellar ataxia^36^. In this study, we investigated the neuroprotective effects of *H. erinaceus* in rat model of 3-acetylpyridine (3-AP)-induced cerebellar ataxia. We hypothesise that *H. erinaceus* rescues the behavioural motor impairments and Purkinje cell deficits in rat model of 3-AP-induced cerebellar ataxia through specific neuroplasticity mechanisms.

## Materials and methods

### Subjects

Male Sprague Dawley rats (n = 38, 10 weeks old) were housed in pairs under controlled conditions at room temperature (25 °C to 27 °C), 60% to 65% humidity, and 12-h light/dark cycle, with food and water provided ad libitum. All experimental procedures and methods were performed in accordance with the relevant guidelines and regulations, and were approved by the Committee on the Use of Live Animals in Teaching and Research (CULATR), The University of Hong Kong (No. 4495–17).

### Extraction and nutritional composition of *H. erinaceus*

A standardised aqueous extract of *H. erinaceus* (NevGro^®^, Batch No. 7H2308X, Ganofarm R&D Sdn Bhd, Tanjung Sepat, Selangor, Malaysia) was used in this experiment. The extract was prepared by boiling fresh fruiting bodies of *H. erinaceus* in reverse osmosis water for 4 h, filtered, concentrated and spray-dried. It contains 20.66% beta 1,3–1,6 glucan and 0.17% adenosine (Nova Laboratories Private Limited, Sepang, Selangor, Malaysia). Total glucan and α-glucan were determined by the β-glucan assay kit (Megazyme International, Wicklow, Ireland). Adenosine content was analysed and quantified by high-performance liquid chromatography (HPLC) using in house method (Nova Laboratories Private Limited, Sepang, Selangor, Malaysia)^37^.

### Experimental design and drug administration

**Figure 1:**
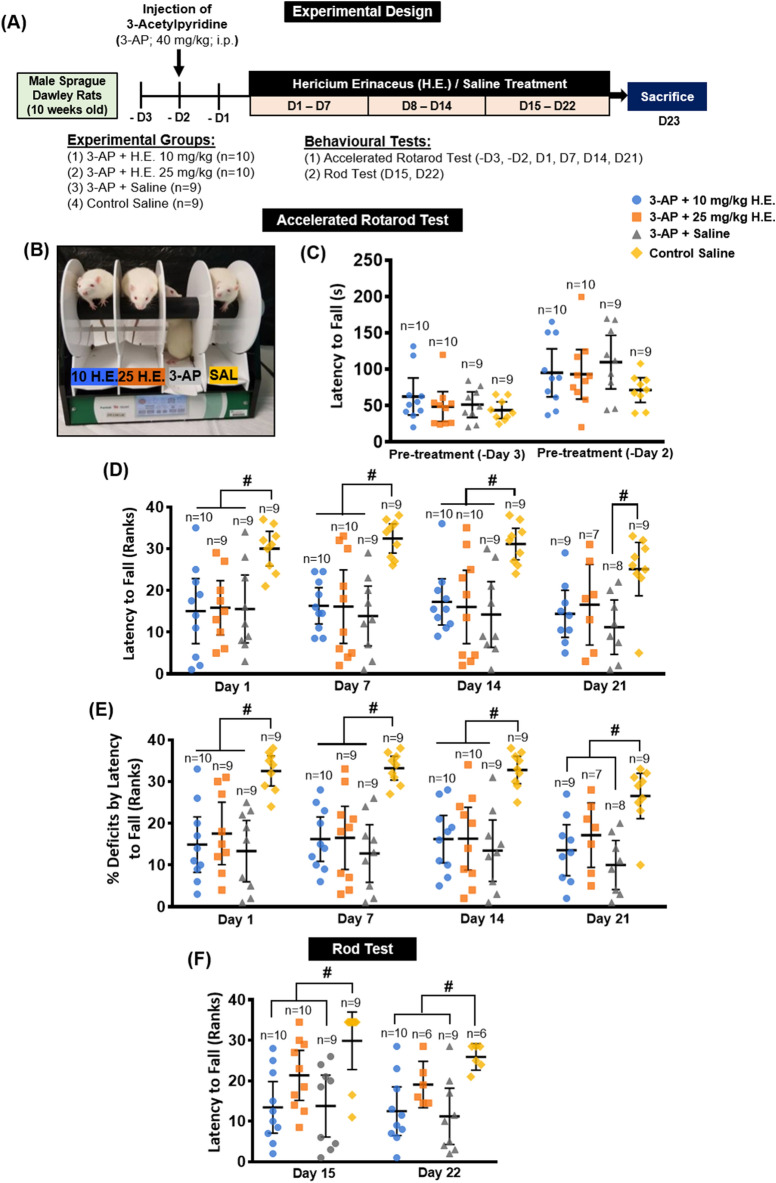
Schematic representation of the experimental design for *H. erinaceus* treatments and a testing of cerebellar ataxia functions in rat model of 3-AP-induced cerebellar ataxia (**A**). Rats from each group were placed on the lane of the rotarod apparatus, respectively (**B**). Rats were trained on the rotarod and baseline performance was assessed on day -3 and day -2 prior to the 3-AP injection (**C**). Accelerated rotarod evaluation of the motor deficits by latency to fall (Rank) (**D**)**,** percentage of deficit (Rank) (**E**), and rod test by latency to fall for the assessment of balance (**F**). The endpoints were rank transformed, and results were presented as mean individual data points with 95% confidence interval. All statistical values have been adjusted by Bonferroni correction for multiple comparisons. Indicators: # Significantly different from the non-3-AP control group. *p* values < 0.05.

For the behavioural baseline assessments, animals were subjected to accelerated rotarod tests on day − 3 and day − 2. Animals were then injected with either 40 mg/kg 3-Acetylpyridine (3-AP; Sigma-Aldrich, Missouri, USA) or saline (0.9% NaCl) on day − 2. After 2 days of 3-AP treatment, all animals were again tested using the accelerated rotarod test. On day 1, the 3-AP-injected animals were administered aqueous extract of *H. erinaceus* intraperitoneally at doses of 10 mg/kg and 25 mg/kg. The control non-3-AP-treated animals and 3-AP-injected animals were both injected with saline. The behavioural battery included accelerated rotarod test, which assesses motor coordination, conducted on days − 3, − 2, 1, 7, 14, and 21; and the rod test, which measures balance and grip strength, conducted on days 15, and 22 (Fig. 1A).

### Behavioural tests

Accelerated Rotarod Test: The test was performed as previously described^38^. Briefly, a rat was placed each of four rods (7 cm lane width) in the Rotarod apparatus (Panlab-Harvard Apparatus, Massachusetts, USA). After four rats were placed into the apparatus, the acceleration program was started, which increased the rotation speed progressively from 4 to 40 revolutions per minute (rpm) (Fig. 1B). The total time spent on the rod (latency to fall) for each rat was recorded. The percentage of deficit was calculated based on the following formula: (X – Baseline) / (X + Baseline) × 100%.

Rod Test: A rod (25 mm diameter) was placed on top of an acrylic box (40 cm high). The rat was placed on the rod along its body length. The time spent balancing on the rod (latency to fall) was recorded for up to 5 min.

### Histological assessment

After the behavioural tests, rats were euthanised with sodium pentobarbital (Dorminal, Alfasan International BV, Woerden, Holland) and decapitated. Rats were perfused with 0.9% saline and brains were removed. Half of the brain was immersed in 4% paraformaldehyde fixative solution for 1 day, followed by 15% and 30% sucrose-buffer solution for cryoprotection prior to freezing. The fixed brain was frozen in liquid nitrogen and stored at − 80 °C for histological studies. The other half of the brain was immediately frozen in liquid nitrogen and stored at − 80 °C for the gene and protein expression study. For the morphological study, the cerebellum was consecutively cryosectioned into 40-μm coronal sections using a cryostat CM1950 (Leica Microsystems, Wetzlar, Germany). Sections were mounted on gelatin-coated slides and stained with haematoxylin and eosin^39^. Morphological changes were examined according to our previously published methods^40^.

### Quantification of Purkinje cells

For the quantification of Purkinje cells, five photomicrographs were randomly acquired using Axiophot2 Imaging Microscope (Carl Zeiss Microscopy GmbH, Gottingen, Germany) at a 40 × magnification, giving a total of 25 photomicrographs for each subject. The number of Purkinje cells visible in each section was counted by two trained volunteers who were blinded to the treatments. The length of Purkinje cell layer in each photomicrograph was measured by drawing a line throughout the centre of the cell bodies of all Purkinje cells in the layer using ImageJ software (National Institutes of Health, Maryland, USA). The number of Purkinje cells and the Purkinje cell layer length were calculated from each of the 25 photomicrographs. Purkinje cell linear density (Purkinje cells per mm of Purkinje cell layer) was calculated by dividing the number of Purkinje cells by the Purkinje cell layer length^41^.

### Real-time PCR

The cerebellum and motor cortex regions were dissected for the neurogenesis- and neuroplasticity-related gene expression study. The real-time PCR was performed according to our previously published methodology^42^. Total RNA was isolated from the cerebellum and motor cortex area using TRIZOL (Life Technologies, Carlsbad, USA) and converted into cDNA using a cDNA synthesis kit (Takara Bio Inc., Shiga, Japan). Quantitative real-time qPCR of neurogenesis- and neuroplasticity-related genes including *doublecortin* (*Dcx*), *nestin* (*Nes*), *brain-derived neurotrophic factor* (*BDNF*), *tropomyosin receptor kinase B (TrkB), cAMP response element binding protein (CREB), postsynaptic density-95 (PSD95), Purkinje cell protein 4 (PCP4), calbindin-D28k*, and *caspase-3* were performed using the StepOnePlus Real-Time PCR system (Thermo Fisher Scientific, Massachusetts, USA) and SYBR Green quantitative PCR mix (Applied Biosystems, Warrington, UK). The primer sequences used can be found in Table 1. Relative expression was calculated as the relative quantification normalised to the reference gene, GADPH, using the ratio 2^-ΔΔC^_T_ method^42,43^.

**Table 1:** The primer sequences used in the real-time quantitative PCR.

Gene	5′–3′ primer sequence
*BDNF*^44^	Forward: TGGCTGACACTTTTGAGCAC Reverse: AAGTGTACAAGTCCGCGTCC
*TrkB*^45^	Forward: CCTCCACGGATGTTGCTGAC Reverse: GCAACATCACCAGCAGGCA
*NeuN*^46^	Forward: GAGGAGTGGCCCGTTCTG Reverse: AGGCGGAGGAGGGTACTG
*PSD95*^47^	Forward: GACGCCAGCGACGAAGAG Reverse: CTCGACCCGCCGTTTG
*Nes*^48^	Forward: AGGCTGAGAACTCTCGCTTGC Reverse: GGTGCTGGTCCTCTGGTATCC
*Dcx*^49^	Forward: ACACCCTTGATGGAAAGCAG Reverse: AGGACCACAAGCAATGAACA
*Caspase-3*^50^	Forward: GTGGAACTGACGATGATATGGC Reverse: CGCAAAGTGACTGGATGAACC
*PCP4*^51^	Forward: CGGAGTCAGGCCAACATGAG Reverse: TGAATGGCCACAGCTGCAC
*Calbindin-D28k*^52^	Forward: ACGGAAGTGGTTACCTGGAA Reverse: CACACATTTTGATTCCCTGG
*GAPDH*^42^	Forward: GTCGGTGTGAACGGATTTG Reverse: AATTTGCCGTGAGTGGAGTC

### Western blot analysis of cerebellar tissue

The cerebellum was homogenised with RIPA buffer containing protease and phosphatase inhibitors (Thermo Scientific, Rockford, Illinois, USA). The protein concentration was measured by Bio-Rad DC Protein Assay Kit (Bio-Rad, Hercules, California, USA). Each sample was separated by 8%–12% SDS-PAGE and transferred to a PVDF membrane (Bio-Rad Laboratories, Hercules, California, USA) using a semi-dry electroblotting system. The membranes were blocked with 5% BSA in TBS-T (20 mM Tris–HCl, 150 mM NaCl, 0.1% Tween 20) for 1 h at room temperature. Blots were incubated at 4 °C overnight with the respective primary antibodies, including ERK1/2, pERK1/2, CREB, pCREB, GAPDH (1:1,000; Cell Signalling Technology, Inc., Beverly, Massachusetts, USA), Syn, (1:2000; Synaptic Systems, Goettingen, Germany) and PSD95 (1:1,000; Abcam, Cambridge, Massachusetts, USA). After adding horseradish peroxidase-conjugated anti-rabbit or anti-mouse immunoglobulin G antibody (Invitrogen, Thermo Fisher Scientific, Massachusetts, USA) for 1 h at room temperature, bound-protein was visualised by chemiluminescence kit (Bio-Rad Laboratories, Inc., Hercules, California, USA). The relative protein expression was normalised against GAPDH.

### Statistical analysis

The results were analysed using IBM SPSS Statistics 25. The normality of data distribution for behavioural endpoints was examined using the Shapiro–Wilk test. Several endpoints exhibited significant departure from normality and the behavioural data for each of these endpoints were rank-transformed, followed by nonparametric Kruskal–Wallis test for intergroup comparisons. All significant values for nonparametric tests were adjusted by Bonferroni correction for multiple comparisons. For normally distributed data, the results were analysed using one-way ANOVA (with repeated-measures) and by Bonferroni *post-hoc* test for multiple planned comparisons, as appropriate. Spearman correlation coefficients with Bonferroni correction were calculated to investigate the relationship between different variables related to cerebellum neuroplasticity genes and/or protein functions, as well as the behavioural measures. Scatter plots were drawn only for specific variables with statistically significant correlations. The methodology of statistical analysis was performed as previously described^53–55^. All results were presented in individual data points with 95% confidence interval, and *p* values < 0.05 were considered significant, as appropriate.

## Results

### Accelerated rotarod test

In the baseline assessment of accelerated rotarod test, repeated-measures ANOVA showed significant main effects for day (F _(1, 34)_ = 36.696, *p* < 0.001), but not group (F _(3, 34)_ = 1.187, *p* = n.s.) and interaction group x day (F _(3, 34)_ = 1.000, *p* = n.s.). One-way ANOVA with Bonferroni *post-hoc* test for multiple comparisons revealed no significance for baseline day − 3 (F _(3, 34)_ = 0.838, *p* = n.s.) and day − 2 (F _(3, 34)_ = 1.238, *p* = n.s.) (Fig. 1C).

Two days after the 3-AP injection on day 1 (before injection of *H. erinaceus*), Kruskal–Wallis test showed significant main effects in animals treated with 3-AP compared to the non-3-AP control animals (H_3_ = 12.309, *p* = 0.006; Fig. 1D). Pairwise comparisons with Bonferroni correction revealed significant impairments in the accelerated rotarod test in all three of the 3-AP animal groups (all *p* < 0.033) compared to the non-3-AP control animals.

In the treatment period of H.E., repeated-measures analysis of accelerated rotarod test showed significant main effects for day (F _(3, 84)_ = 3.240, *p* = 0.026), and group (F _(3, 28)_ = 9.203, *p* < 0.001), but not for interaction (F _(9, 84)_ = 1.079, *p* = n.s.). Kruskal–Wallis tests revealed statistically significant differences on day 7 (H_3_ = 16.276, *p* = 0.001), day 14 (H_3_ = 13.239, *p* = 0.004), and day 21 (H_3_ = 9.903, *p* = 0.019). On days 7 and 14, comparisons with Bonferroni correction showed significant decreases of latency to fall in 3-AP animals treated with 10 mg/kg and 25 mg/kg, or saline compared to non-3-AP control group (all *p* < 0.04). On day 21, there were no statistically significant differences between 3-AP + 10 mg/kg H.E. and 3-AP + 25 mg/kg H.E. groups compared to the 3-AP + saline group (*p* = n.s.). Interestingly, our results also showed no differences for 3-AP + 10 mg/kg H.E. and 3-AP + 25 mg/kg H.E. groups compared to the non-3-AP control (*p* = n.s.), indicating a potential rescue of locomotor abnormalities in 3-AP-induced animals with cerebellar ataxia. However, there was a statistically significant difference in the 3-AP + saline group compared to the non-3-AP control (*p* = 0.018).

### Percentage of deficits by latency to fall

To accurately compare the results of the accelerated rotarod over the duration of treatment, the changes in percentage of deficits by latency to fall were normalised according to the respective individual animal’s baseline on pre-treatment day − 2. Repeated-measures analysis showed significant main effects for day (F _(3, 84)_ = 3.883, *p* = 0.012) and group (F _(3, 28)_ = 13.712, *p* < 0.001), but not for interaction (F _(9, 84)_ = 1.015, *p* = n.s.). Nonparametric Kruskal–Wallis tests revealed statistically significant differences on day 1 (H_3_ = 16.695, *p* = 0.001), day 7 (H_3_ = 18.626, *p* < 0.001), day 14 (H_3_ = 17.231, *p* = 0.001), and day 21 (H_3_ = 14.125, *p* = 0.003). On days 1, 7 and 14, comparisons with Bonferroni correction showed remarkable decreases in the percentage of motor deficits by latency to fall in 3-AP animals treated with 10 mg/kg H.E., 25 mg/kg H.E., and saline compared to non-3-AP control group (all *p* < 0.030; Fig. 1E). On day 21, we found significant differences for 3-AP + 10 mg/kg H.E. (*p* = 0.026) and 3-AP + saline (*p* = 0.003) groups but not for 3-AP + 25 mg/kg H.E. group (*p* = 0.320) compared to the non-3-AP control animals, again suggesting 25 mg/kg H.E. treatment has potential neuroprotective effects in cerebellar ataxia.

### Rod test

In the rod test, repeated-measures analysis showed significant main effects for day (F _(1, 29)_ = 11.469, *p* = 0.002) and group (F _(3, 29)_ = 5.770, *p* = 0.003), but not for interaction (F _(3, 29)_ = 1.171, *p* = n.s.). Nonparametric Kruskal–Wallis tests revealed statistically significant differences on day 15 (H_3_ = 11.803, *p* = 0.008) and day 22 (H_3_ = 10.387, *p* = 0.016). On days 15 and 22, comparisons with Bonferroni correction showed significant impairment of motor coordination and balance performance in the 3-AP + saline and 3-AP + 10 mg/kg H.E. groups compared to the non-3-AP control group. Although no remarkable difference was observed between 3-AP + 25 mg/kg H.E. and 3-AP + saline group, we found that animals in 3-AP + 25 mg/kg H.E. group showed no difference when compared to the non-3-AP control group (Day 15: H_3_ =  − 8.539, *p* = 0.557; Day 25: H_3_ =  − 6.833, *p* = 1.000; Fig. 1F), indicating a potential recovery of motor coordination and balance in the 3-AP + 25 mg/kg H.E. group.

### *Hericium erinaceus* (25 mg/kg) treatment normalised neuroplasticity-related genes

**Figure 2:**
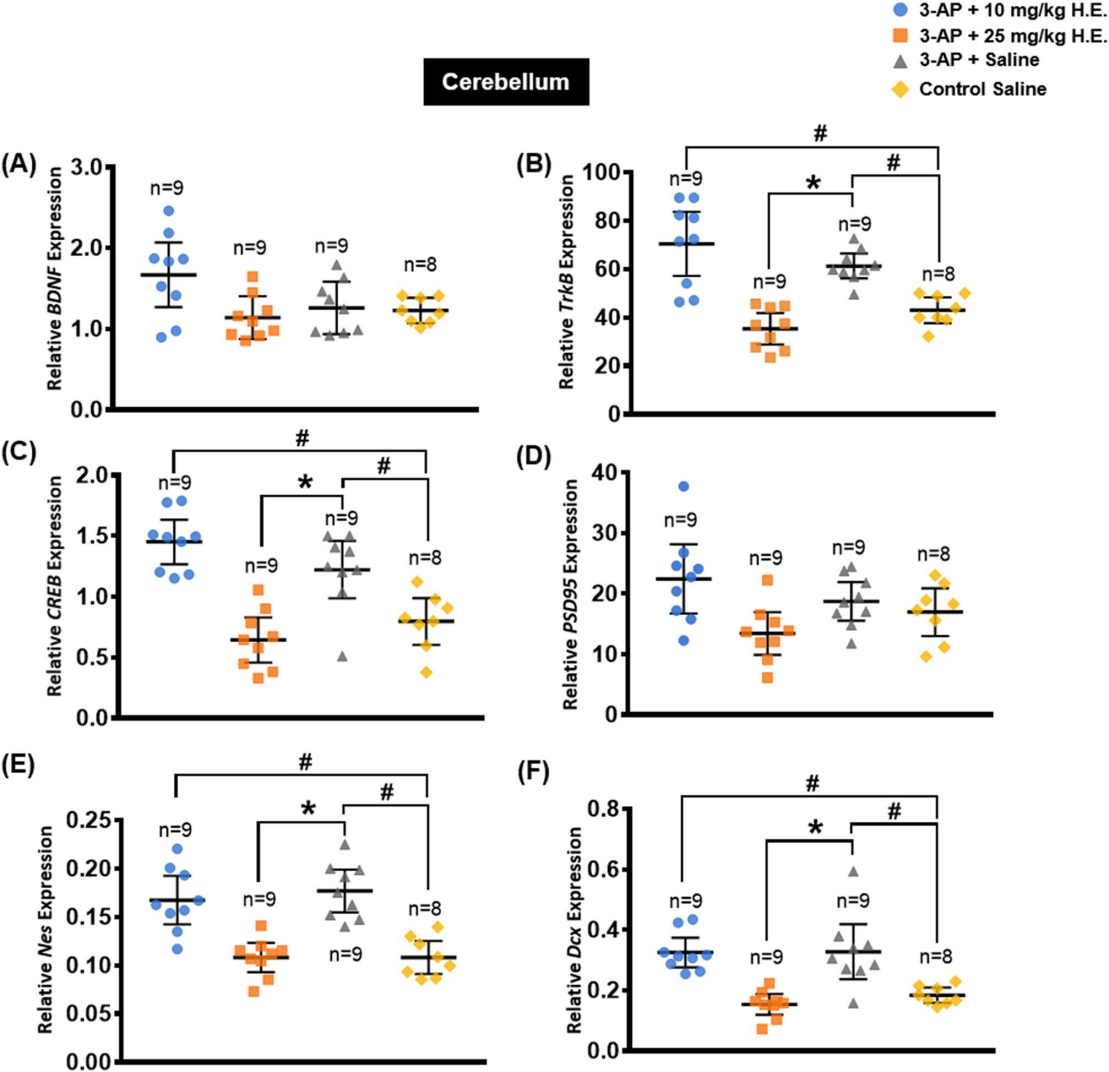
Effects of 3-AP and *H. erinaceus* on the relative expression of neuroplasticity- and neurogenesis-related genes including *BDNF* (**A**), *TrkB* (**B**), *CREB* (**C**), *PSD95* (**D**), *Nes* (**E**), *Dcx* (**F**) in the cerebellum of 3-AP + 10 mg/kg H.E., 3-AP + 25 mg/kg H.E., 3-AP + saline, and non-3-AP control groups. *TrkB, CREB, Nes,* and *Dcx* were significantly upregulated in 3-AP-induced ataxic rats without *H. erinaceus* treatment. Notably, 25 mg/kg *H. erinaceus* normalised the neuroplasticity-related gene expressions to the levels in the non-3-AP control. Relative expression was calculated by normalising the relative quantifications to the reference gene GAPDH as the ratio of the 2^^CT(reference)^ and 2^^CT(test)^. Indicators: # Significantly different from the non-3-AP control group. * Significantly different from the 3-AP + saline group. *p* values < 0.05.

**Figure 3:**
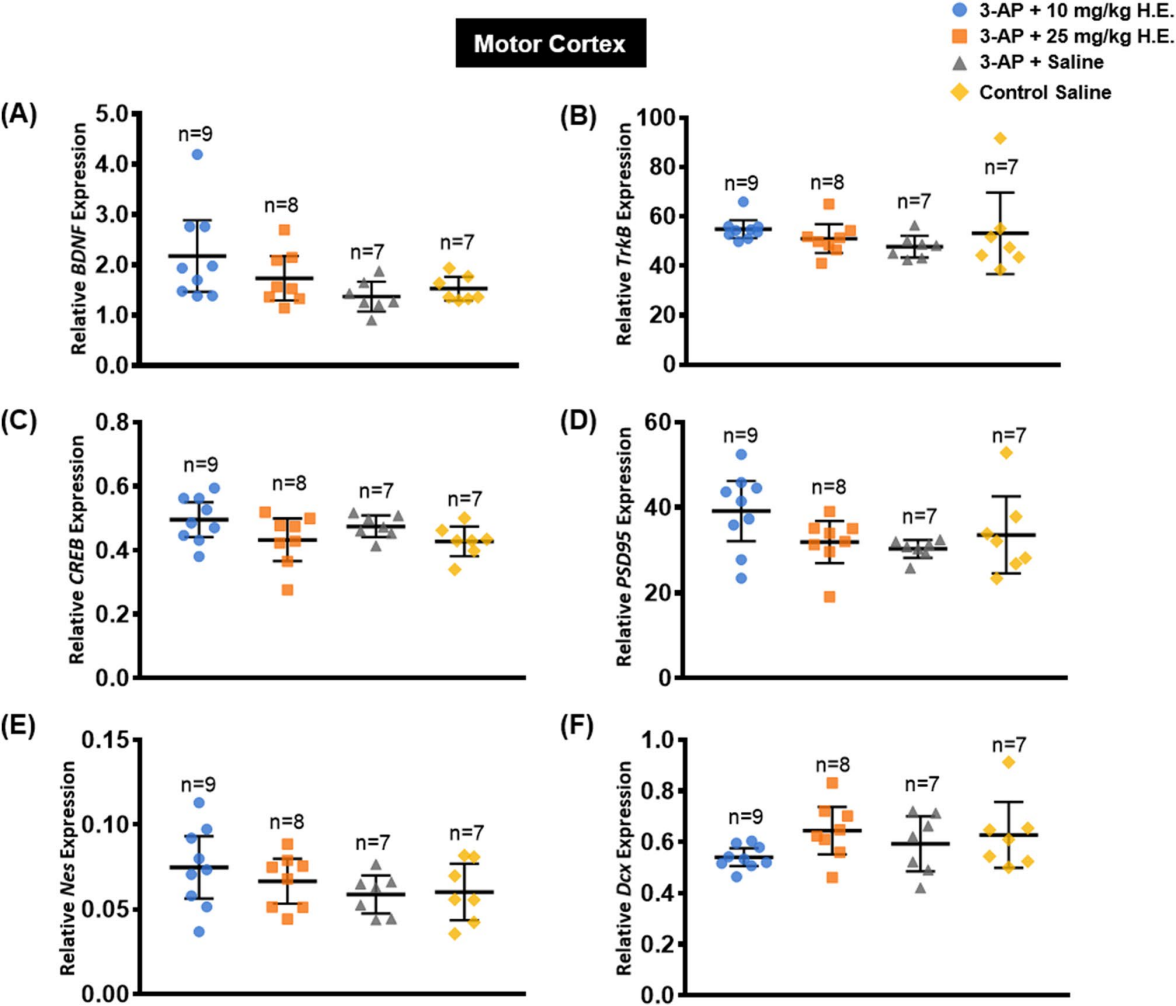
Effects of 3-AP injection and *H. erinaceus* treatment on the expression of neuroplasticity- and neurogenesis-related genes including *BDNF* (**A**), *TrkB* (**B**), *CREB* (**C**), *PSD95* (**D**), *Nes* (**E**), and *Dcx* (**F**) in the motor cortex of 3-AP + 10 mg/kg H.E., 3-AP + 25 mg/kg H.E., 3-AP + saline, and non-3-AP control groups. No significant changes were observed in the expression of neuroplasticity- and neurogenesis-related genes in the motor cortex among the different groups. Relative expression was calculated by normalising the relative quantifications to the reference gene GAPDH as the ratio of the 2^^CT(reference)^ and 2^^CT(test)^. Indicators: * Significantly different from the non-3-AP control group. # Significantly different from the 3-AP + saline group. *p* values < 0.05.

In the cerebellum, one-way ANOVA showed significant effects on the expression of *BDNF* (F _(3, 31)_ = 4.123, *p* = 0.014) (Fig. 2A), *TrkB* (F _(3, 31)_ = 20.070, *p* < 0.001) (Fig. 2B), *CREB* (F _(3, 31)_ = 18.756, *p* < 0.001) (Fig. 2C), *PSD95* (F _(3, 31)_ = 4.324, *p* < 0.012) (Fig. 2D), *Nestin* (F _(3, 31)_ = 17.683, *p* < 0.001) (Fig. 2E), and *Dcx* (F _(3, 31)_ = 13.806, *p* < 0.001) (Fig. 2F). Interestingly, multiple comparisons with Bonferroni *post-hoc* test demonstrated significant increases in the gene expression of *TrkB*, *CREB*, *Nes,* and *Dcx* in the 3-AP + saline and 3-AP + 10 mg/kg H.E. groups compared to the non-3-AP control group (all *p* < 0.036). Although we found no significant differences in the expressions of *TrkB, CREB, Nestin,* and *Dcx* in 3-AP + 25 mg/kg H.E. group compared to the non-3-AP control group, there were remarkable differences in the expressions of these gene when compared to 3-AP + saline group (all *p* < 0.001). In the motor cortex, we found no significant differences in the gene expression of *BDNF, TrkB, CREB, PSD95, Nestin,* and *Dcx* (all F _(3, 27)_ < 2.746, *p* = n.s.; Fig. 3).

### *Hericium erinaceus* (25 mg/kg) increased the expression of neuroplasticity-related proteins

**Figure 4:**
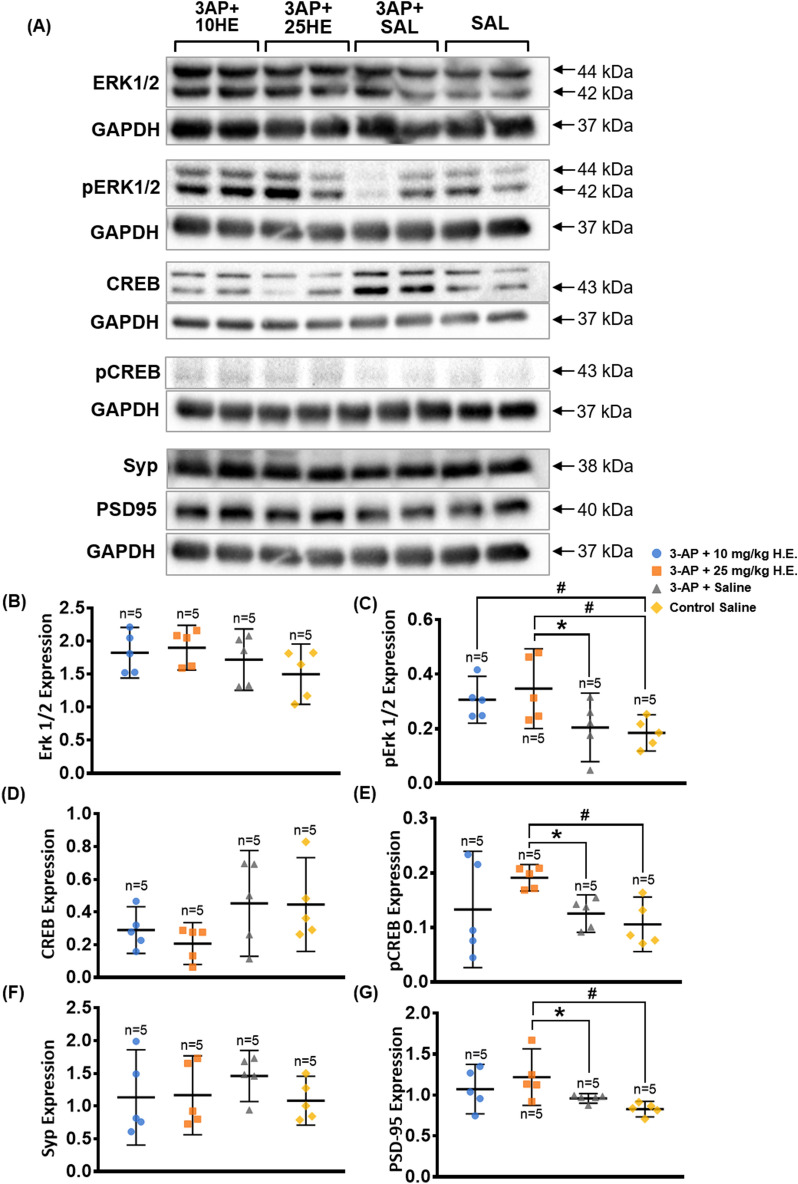
Western blot analysis of neuroplasticity-related proteins in cerebellar tissue from rats administered with 3-AP + 10 mg/kg H.E., 3-AP + 25 mg/kg H.E., 3-AP + saline and saline only (A). Note, dividing lines and white space representing the blots were cropped from different blots. Graphical representation of the effects of 3-AP and *H. erinaceus* administration on the expression of neuroplasticity-related proteins (**A**) including ERK1/2 (**B**), pERK1/2 (**C**), CREB (**D**), pCREB (**E**), Syp (**F**), and PSD95 (**G**). The expression of the target protein was normalised to the expression of GAPDH. Note, there were significant increases in the protein expressions of pERK1/2, pCREB and PSD95 in 3-AP + 25 mg/kg H.E. group compared to both the 3-AP + saline group and non-3-AP control group. Indicators: * Significantly different from the non-3-AP control group. # Significantly different from the 3-AP + saline group. *p* values < 0.05.

The aqueous extract of *H. erinaceus* used in this study has been standardised to contain 20.66% beta 1,3–1,6 glucan and 0.17% adenosine^37^, and these compounds have been shown to play pivotal roles in neuroprotection and neuroplasticity-related functions^56,57^. In this study, we focused primarily on the neuroplasticity-related mechanisms of ERK-CREB-PSD95 (Fig. 4A), and our results with one-way ANOVA revealed significant differences in the protein expression of pERK1/2 (F _(3, 16)_ = 3.860, *p* = 0.030) (Fig. 4C), PSD95 (F _(3, 16)_ = 3.807, *p* = 0.031) (Fig. 4G), and a marginal difference for pCREB (F _(3, 16)_ = 2.643, *p* = 0.085) (Fig. 4E) in the cerebellum, while no significant difference were found in the protein expression of ERK1/2 (Fig. 4B), CREB (Fig. 4D) and Syp (Fig. 4F) (*p* = n.s.). Interestingly, planned comparison showed significantly higher levels of protein expression for pERK1/2, pCREB, and PSD95 in 3-AP + 25 mg/kg H.E. group compared to the 3-AP + saline group (*t*_(16)_ = 2.525, *p* = 0.023; *t*_(7.223)_ = 4.307, *p* = 0.003; *t*_(16)_ = 2.159, *p* = 0.046; respectively) and non-3-AP control group (*t*_(16)_ = 2.878, *p* = 0.011; *t*_(5.813)_ = 4.266, *p* = 0.006; *t*_(16)_ = 3.245, *p* = 0.005; respectively). A significant difference in the expression level of pERK1/2 was also found between 3-AP + 10 mg/kg H.E. and non-3-AP control group (*t*_(16)_ = 2.150, *p* = 0.047).

### *Hericium erinaceus* (25 mg/kg) rescued the degeneration of Purkinje cells

**Figure 5:**
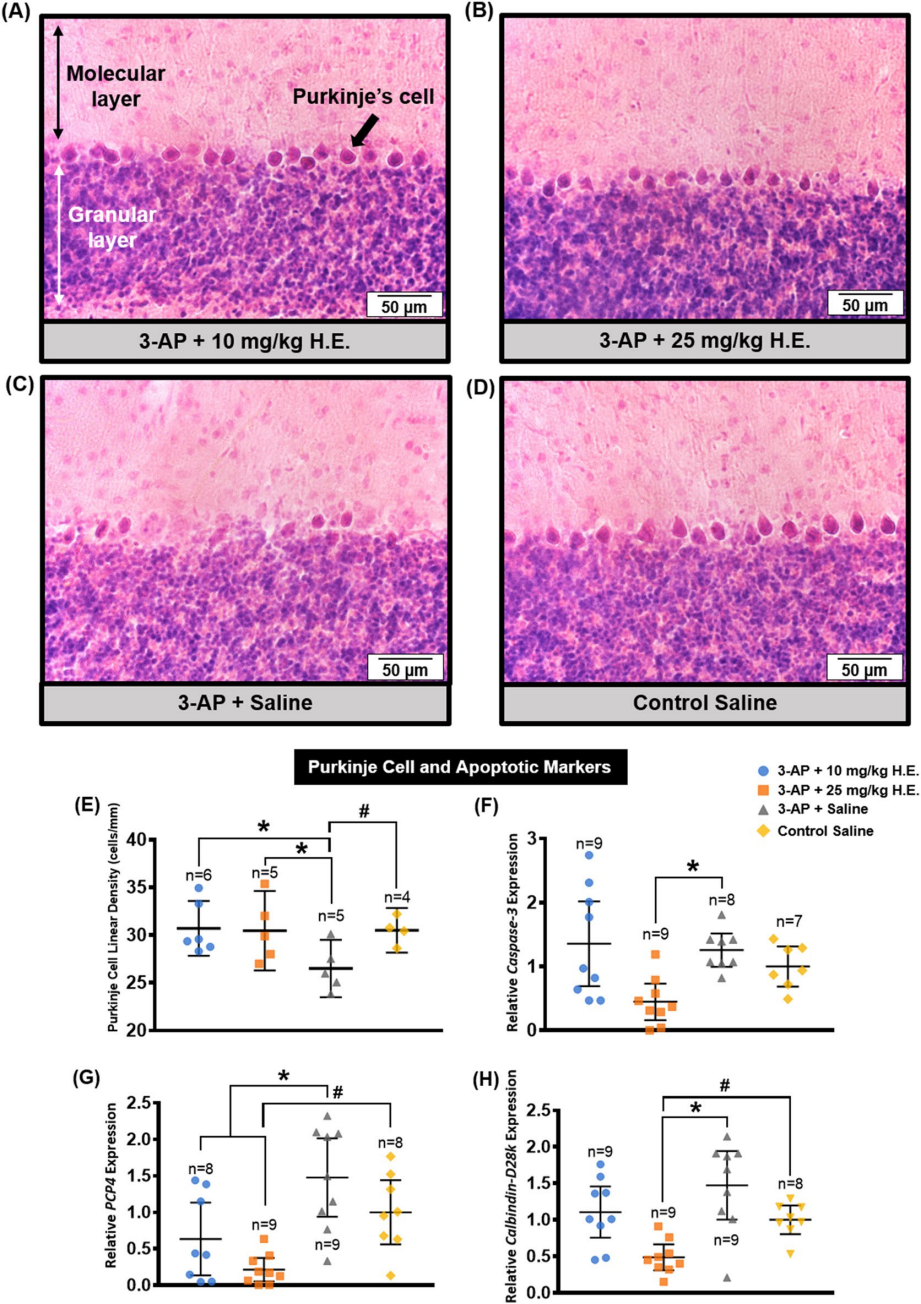
Effects of 3-AP and *H. erinaceus* administration on the morphological changes of Purkinje cells by haematoxylin and eosin staining. Notably, animals in the 3-AP + 10 mg/kg H.E. (**A**) and 3-AP + 25 mg/kg H.E. groups (**B**) showed Purkinje cells with regular and ordinary morphology that was similar to the non-3-AP control group. Interestingly, Purkinje cells in the 3-AP + saline group (**C**) were scattered with disturbed alignment and irregular appearance. The Purkinje cells in the non-3-AP control group (**D**) showed regular morphology and ordinary alignment between granular and molecular layers. The scale bars represent 50 μm. The Purkinje cell linear density of 3-AP + 10 mg/kg H.E. (1752 Purkinje cells per 58,729.942 μm total PC length, 30 sections from 6 animals), 3-AP + 25 mg/kg H.E. groups (1519 Purkinje cells per 50,799.906 μm total PC length, 25 sections from 5 animals), and the non-3-AP control group (1,295 Purkinje cells per 42,788.036 μm total PC length, 20 sections from 4 animals) showed significant differences compared to 3-AP + saline group (1,348 Purkinje cells per 51,171.87 μm total PC length, 25 sections from 5 animals) (**E**). Interestingly, there was a remarkable reduction of *caspase-3* in 3-AP + 25 mg/kg H.E. animals compared to the 3-AP + saline group, indicating the neuroprotective effects of 25 mg/kg of *H. erinaceus* against apoptosis (**F**). A significant decrease of gene expression for *PCP4* (**G**) and *calbindin-D28k* (**H**) was found in 3-AP + 25 mg/kg H.E. group compared to the 3-AP + saline and the non-3-AP control groups. Indicators: * Significantly different from the non-3-AP control group. # Significantly different from the 3-AP + saline group. *p* values < 0.05.

A row of uniformly aligned Purkinje cells was present between the relatively lightly-stained molecular layer and the darkly-stained granular layer in the non-3-AP control group (Fig. 5D). However, some of the Purkinje cells in 3-AP + saline group (Fig. 5C) exhibited a disturbed alignment of Purkinje cells with deformed, irregular, and scattered appearance. Interestingly, 3-AP + 10 mg/kg H.E. (Fig. 5A) and 3-AP + 25 mg/kg H.E. (Fig. 5B) groups showed aligned Purkinje cells with regular and ordinary morphology, which was relatively indistinguishable from the non-3-AP control group. Comparison analysis revealed that the Purkinje cell linear density of 3-AP + saline group (1,348 Purkinje cells per 51,171.87 μm total PC length, 25 sections from 5 animals) was significantly lower compared to the non-3-AP control group (1,295 Purkinje cells per 42,788.036 μm total PC length, 20 sections from 4 animals) (*t*_(16)_ = -2.264, *p* = 0.038) (Fig. 5E). Interestingly, animals receiving 10 mg/kg (1752 Purkinje cells per 58,729.942 μm total PC length, 30 sections from 6 animals) and 25 mg/kg H.E. (1519 Purkinje cells per 50,799.906 μm total PC length, 25 sections from 5 animals) treatments showed no significant differences compared to the non-3-AP control group (*t*_(16)_ < 0.124, *p* = n.s), but they were significantly different from the 3-AP + saline group (*t*_(16)_ = 2.640, *p* = 0.018 ; *t*_(16)_ = 2.373, *p* = 0.031, respectively).

In support of the histological findings, qPCR on transcription factors (*calbindin-D28k* and *PCP4*) that are highly specific to the postmitotic neurons of Purkinje cell was performed. One-way ANOVA showed main effects on the gene expression of *calbindin-D28k* (F _(3, 32)_ = 9.009, *p* < 0.000) and *PCP4* (F _(3, 33)_ = 8.878, *p* < 0.000) in the cerebellum. Interestingly, we found a significant decrease of gene expression for *PCP4* and *calbindin-D28k* in 3-AP + 25 mg/kg H.E. group compared to the 3-AP + saline and the non-3-AP control groups (all *p* < 0.001; Fig. 5G,H). Besides, there was also a remarkable decrease of *PCP4* gene expression in 3-AP + 10 mg/kg H.E. group compared to the 3-AP + saline group (*p* = 0.018). The reduction of these transcription factors could possibly be explained by which the RNA was used to synthesise calbindin-D28k and PCP4 proteins for gain-of-function purpose in the 3-AP + 25 mg/kg H.E. treated animals. To further investigate the crucial function of apoptosis played by *H. erinaceus* treatment in this ataxic model, our result show a significant main effect for *caspase-3* (F _(3, 31)_ = 5.978, *p* = 0.003), and Bonferroni post-hoc test for multiple comparisons demonstrated a remarkable reduction of *caspase-3* in 3-AP + 25 mg/kg H.E. animals compared to the 3-AP + saline group (*p* = 0.012), indicating the neuroprotective effects of 25 mg/kg of *H. erinaceus* against apoptosis induced by 3-AP (Fig. 5F).

### Correlations between the cerebellar neuroplasticity-related functions and the behavioural performances

**Figure 6:**
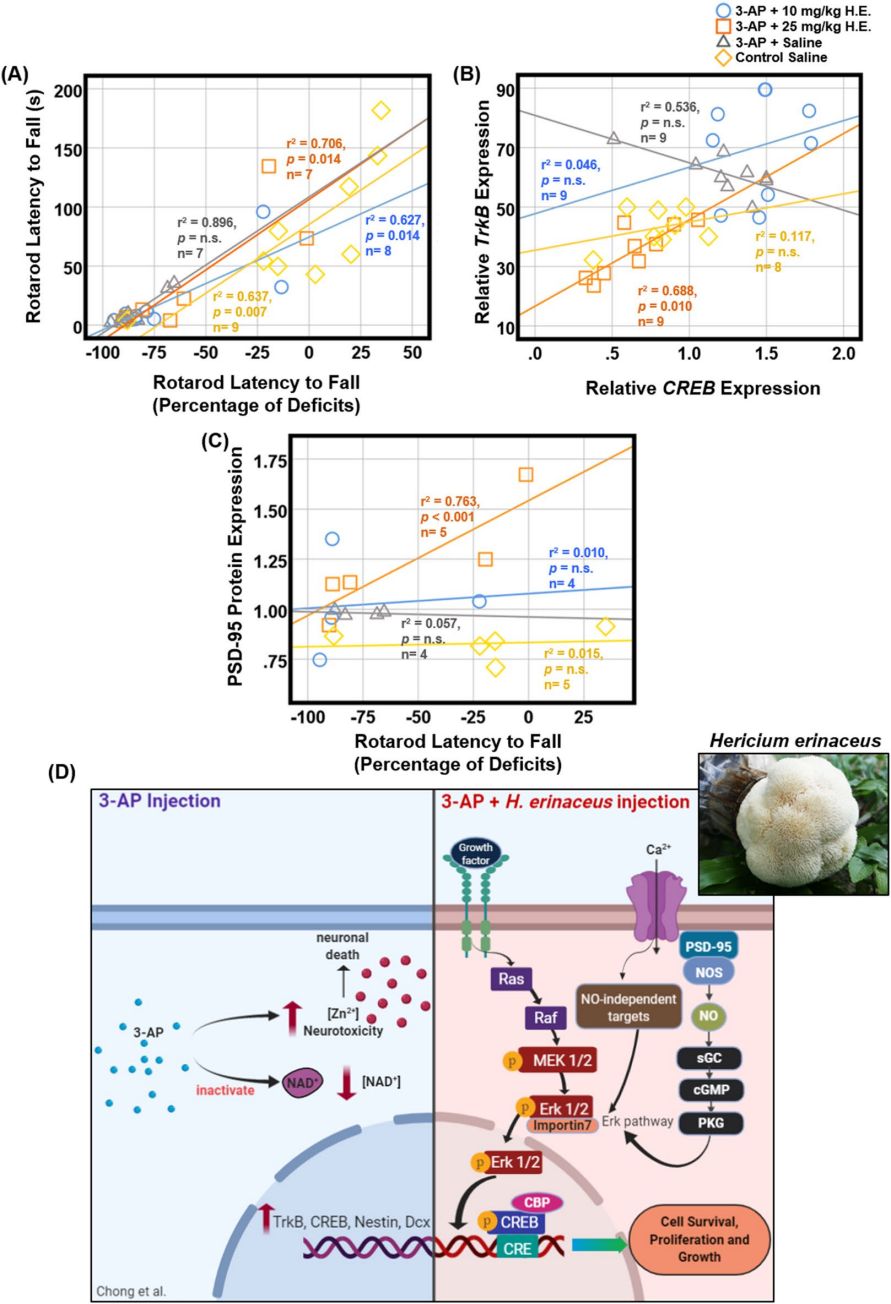
Scatter plots displaying the correlations between the variables related to the motor behavioural tests, as well as the cerebellar neuroplasticity- and neurogenesis-related relative gene and protein expressions. Notably, there were significant correlations between the accelerated rotarod behavioural data and the percentage of deficits in 3-AP + 10 mg/kg H.E., 3-AP + 25 mg/kg H.E., and non-3-AP control groups. No correlation was found between the accelerated rotarod behavioural data and the percentage of deficits in 3-AP + saline group (r^2^ = 0.896, *p* = n.s.), suggesting the behavioural motor coordination of accelerated rotarod was altered by 3-AP injection (**A**). In the 3-AP + 25 mg/kg H.E. group, the gene expression of *TrkB* was positively correlated with the *CREB* expression (**B**), and the PSD95 protein expression was positively correlated with the percentage of deficits by latency to fall (**C**). The hypothetical mechanism of neuronal cell death induced by 3-AP (left) and pathways of *H. erinaceus* on the role of pERK1/2-pCREB-PSD95 in neuroplasticity-related mechanisms (right). 3-AP reduces the intracellular concentration of NAD^+^ and interferes with Zn^2+^ homeostasis, which increases the Zn^2+^ neurotoxicity and lead to neuronal death. Upregulation of neuroplasticity and neurogenesis-related genes including *TrkB, CREB, Nestin,* and *Dcx* was stimulated by 3-AP to reverse neuronal death (left). After the injection of 3-AP followed by *H. erinaceus,* the tyrosine kinase receptors are hypothetically activated by NGF and followed by the recruitment of the cytoplasmic protein, Son of Sevenless (SOS). This triggers the activation of the Ras/guanosine triphosphate complex^86^ and initiates cytoplasmic kinase signal transduction cascades^87^. MEK1*/*2 catalyses the phosphorylation of ERK1*/*2 at Tyr204/187 mediated by importin-7, followed by translocation from the cytoplasm to the nucleus^88,89^. ERK1/2 acts as an upstream regulator by catalysing the phosphorylation of various cytoplasmic and nuclear substrates that encode transcription factors and gene regulatory proteins, including CREB^87,90^. Additionally, the Ca^2+^ influx is through the NMDA receptors, which activates the neuronal nitric oxide synthase, that is mediated by PSD-95^80,81^. The induced nitric oxide activates ERK for the expression of neuroplasticity-related proteins, facilitated by cGMP and PKG (right)^82^. This hypothetical model was created by BioRender.com (**D**).

Spearman correlation analysis showed significant correlation between the accelerated rotarod behaviour data and the percentage of deficit in the 3-AP + 10 mg/kg H.E. (r^2^ = 0.627, *p* = 0.014), 3-AP + 25 mg/kg H.E. (r^2^ = 0.706, *p* = 0.014) and the non-3-AP control group (r^2^ = 0.637, *p* = 0.007), indicating a potential restoration of motor coordination impairment in the 3-AP + 10 mg/kg and 25 mg/kg H.E. groups compared to the normal motor functions of non-3-AP control animals (Fig. 6A; Table 2). No correlation was found between the accelerated rotarod behaviour data and the percentage of deficit in 3-AP + saline group (r^2^ = 0.896, *p* = n.s.), suggesting the motor coordination behaviour in the accelerated rotarod was altered by 3-AP injection. Interestingly, we found a significant positive correlation between the gene expression for *CREB* and *TrkB* in the 3-AP + 25 mg/kg H.E. group (r^2^ = 0.688 *p* = 0.010), indicating a close link between these two genes in the induced neuroplasticity in the cerebellum after H.E. injection (Fig. 6B). Furthermore, significant correlations were also found between the PSD95 protein expression and the rotarod percentage of deficit by latency to fall (r^2^ = 0.344, *p* < 0.001) in 3-AP + 25 mg/kg H.E. group (Fig. 6C).

**Table 2:**
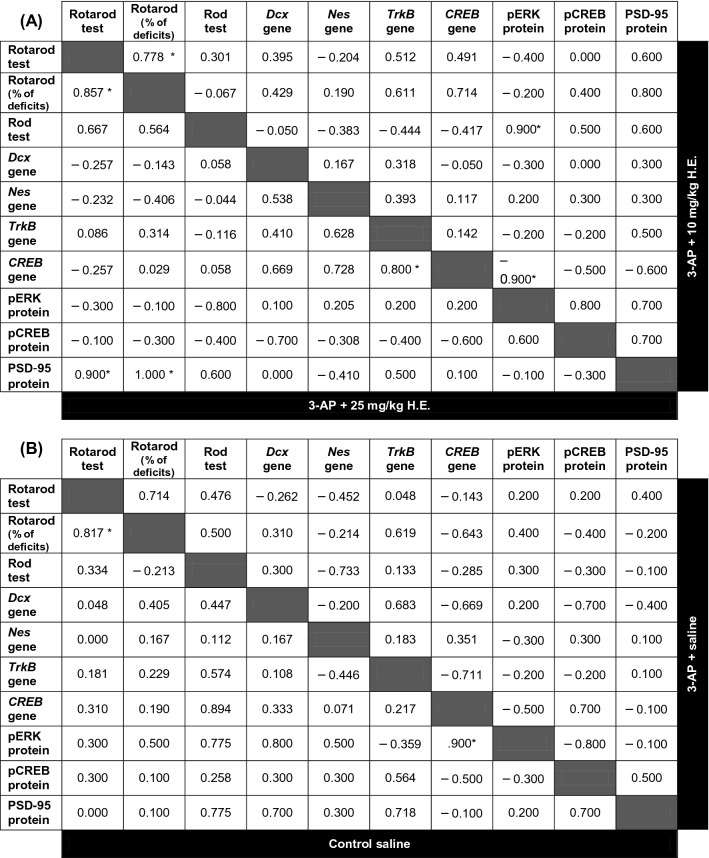
The tables show Spearman’s rho correlation coefficients between variables related to the motor behaviours, neuroplasticity- and neurogenesis-related genes, and protein expressions after administration of *H. erinaceus* in the animal model of 3-AP-induced cerebellar ataxia. The tables show the correlations of the 3-AP + 10 mg/kg H.E. group in the upper-right side and 3-AP + 25 mg/kg H.E. group in the lower-left side (**A**), and 3-AP + saline group in the upper-right side and non-3-AP control group in the lower-left side (**B**). For the statistical analysis, all *p* values were adjusted by Bonferroni correction for multiple comparisons. Indication: *correlation is significant at *p* < 0.0167 for behavioural and protein expression data, and *p* < 0.0125 for gene expression data, respectively.

## Discussion

In the initial experimental design, a dose of 65 mg/kg 3-AP was selected to generate the animal model of cerebellar ataxia based on previous studies^58,59^. Surprisingly, all rats administered 65 mg/kg 3-AP injection died within 24 h. We conducted a pilot study to establish the optimal dose for generating the ataxic animal model using lower doses of 40 and 50 mg/kg 3-AP. Our results showed that animals administered 50 mg/kg 3-AP injection still died within 5–7 days, whereas animals injected with 40 mg/kg 3-AP survived and were accompanied by impairment of behavioural motor coordination and balance (Fig. 1D–F). Therefore, 40 mg/kg 3-AP injection was used in the present study to test the hypothesis of the neuroprotective effects of *H. erinaceus* in rescuing the behavioural motor deficits in 3-AP-induced cerebellar ataxia.

In this study, animals were trained on an accelerated rotarod on day − 3 and day − 2, and their baseline levels were assessed to ensure that all groups possessed comparable motor function behaviour before induction of cerebellar ataxia. Two days after the 3-AP injection on day 1, animals were subjected to the accelerated rotarod test to examine the effectiveness of 3-AP to induce ataxia. Our statistical analysis using Shapiro–Wilk test demonstrated the behavioural data for accelerated rotarod (days 1, 7, 14, and 21) and rotarod (days 15 and 22) tests were not normally distributed; and therefore, the behavioural results were rank transformed and analysed by nonparametric tests according to our previously reported statistical methodology^53^. Our results demonstrated the neurotoxin 3-AP induced remarkable motor impairments in the accelerated rotarod and rod tests compared to the non-3-AP control animals. After *H. erinaceus* treatments, we found no significant improvements on the behavioural motor deficits in the 3-AP + 10 mg/kg and 25 mg/kg H.E. groups compared to the 3-AP + saline group. However, we also observed no remarkable differences in the motor impairment of the 3-AP + 25 mg/kg H.E. group compared to the non-3-AP control group on day 21, indicating a potential recovery of motor deficits by higher dose *H. erinaceus* treatment in this ataxic model. There are limitations in the present study in that no significant effects were observed for H.E. on rescuing the behavioural motor impairments compared to the 3-AP + saline group. Some possible explanations could be the short duration of treatment, as the H.E. injections were administered over 3 weeks, or a higher dosage (e.g., 50 mg/kg) may be required to rescue the behavioural motor deficits. In this study, we have ruled out the possibility of a higher dosage requirement to rescue the behavioural motor deficits. Our unpublished data showed that animals injected with 50 mg/kg H.E. became very sick with significant weight reduction after the second week of injection. Therefore, these animals were humanely euthanised based on the regulations of CULATR and the Association for Assessment and Accreditation of Laboratory Animal Care. The unfavourable outcome in the animals administered 50 mg/kg H.E. was possibly due to toxicity or overdose. This leaves the possibility that prolonged treatment (> 3 weeks) might be needed to observe the effectiveness of *H. erinaceus* in rescuing the motor impairments. However, the animal model of 3-AP-induced cerebellar ataxia used in the present study has been shown to have temporal behavioural motor deficits, in which the ataxic animals recovered from the neurotoxic effects of 3-AP over an extended experimental period^60^. It has been reported that adult rats with 3-AP-induced motor disturbances recovered from the ataxia with less debilitating abnormal movements at 28–43 days after the 3-AP injection^60^. Given that this 3-AP-induced ataxic model has been widely used in many studies^58,59^, the limitation of temporal motor impairments should be taken into consideration when assessing motor function in this model. In view of this, an alternative approach using a genetic model of cerebellar ataxia may be appropriate to assess the neuroprotective function of *H. erinaceus*.

To further investigate the underlying neuroprotective mechanism of *H. erinaceus*, gene expression and protein studies of the effects on neuroplasticity were performed. We found significant increases in neuroplasticity and neurogenesis-related gene expressions of *Dcx, Nes, TrkB,* and *CREB* in both the 3-AP + saline and 3-AP + 10 mg/kg H.E groups. The upregulation of gene expression in these groups could be triggered by the activation of molecular recovery mechanisms upon neuronal death induced by the neurotoxin 3-AP, suggesting a compensatory neuronal regeneration or sprouting of injured axons. This observation was in line with a previous study that showed there were regulatory mechanisms in neural stem cells in injured brain as indicated by increased expressions of *Dcx* and *Nes*^61^. Adult neural stem cells can be regulated by various stimuli, and can undergo proliferation as well as differentiation through neurogenesis to replace the damaged or lost neurons^62^. Although stroke-induced neurogenesis has been reported^63,64^, neural stem cells have limited capacity for neurogenesis, which tends to decrease with age as observed in the brains of older patients with stroke^65^. Hence, full recovery from brain injury may not be achievable through neurogenesis. Neurogenesis mechanisms may be activated through upregulating the neurogenesis-related genes after the 3-AP injection in order to compensate the cells lost resulted by the 3-AP neurotoxin. However, the compensatory mechanism might not be sufficiently effective in regenerating mature neurons and recovering the cerebellum, as well as recovering the motor behavioural deficits. In contrast, ataxic rats that received 25 mg/kg H.E. treatment for 3 weeks did not show increases in neuroplasticity and neurogenesis-related gene expressions, with the expressions similar to that of the non-3-AP control. This phenomenon could be resulted from the stronger neuroprotective effects of a higher dose of *H. erinaceus,* which protected or rescued the cells in the cerebellum from being damaged by 3-AP, and thus the upregulation of the neurogenesis-related genes could be inessential. Our findings suggest that the higher dose of *H. erinaceus* could possibly rescue motor deficits in 3-AP-induced cerebellar ataxia. In this study, we found no significant differences in the gene expressions in the motor cortex, implying that injection of 3-AP was specifically affecting the cerebellar cortex, particularly the Purkinje cells and other cerebellar morphology^66–68^.

To support the findings of gene expression study, Western blot analysis was carried out to investigate protein changes related to neuroplasticity function. We found remarkable increases in pERK1/2, pCREB, and PSD95 protein expression levels in 3-AP + 25 mg/kg H.E. group compared to both the 3-AP + saline group and non-3-AP control group. The increased protein expression levels in 3-AP + 25 mg/kg H.E. group was possibly due to compensatory regenerative mechanisms related to the neuronal loss induced by the neurotoxic effects of 3-AP. In this study, we found no significant changes in neuroplasticity-related proteins between the 3-AP + saline group and non-3-AP control group, indicating the expression levels of ERK-CREB-PSD95 in the non-3-AP control were normal in healthy animals. The results suggest the neuroprotective effects of *H. erinaceus* could be mediated by the protein expression of pERK1/2, pCREB and PSD95, leading to the enhancement of fundamental neuroplasticity processes including neuronal survival and proliferation, and ultimately rescuing the behavioural motor deficits.

In the histological study, we observed the Purkinje cells had regular morphology with normal alignment in the cell layer of the cerebellar cortex in animals treated with *H. erinaceus* (10 mg/kg and 25 mg/kg) and non-3-AP control group. However, ataxic rats without *H. erinaceus* treatment had Purkinje cells with abnormal morphological features of distorted cell bodies and loss of cell continuity (random spatial arrangement at wider distances) in the cerebellar cortex, indicating 3-AP exerted its toxic effects by disrupting the Purkinje cell and/or the spatial distribution of Purkinje cells in the cerebellum. This observation was in line with the findings by Mohammadi and colleagues, in which animals treated with 3-AP injection exhibited Purkinje cells with random arrangement at larger distances compared to the non-3AP control group^68^. Purkinje cells are the sole output neurons in the cerebellum that receive and integrate inputs from parallel fibres and climbing fibres for motor coordination and balance. Neurotoxic 3-AP causes distinctive lesions in the central nervous system by destroying inferior olivary nuclei and their climbing fibres that synapse with Purkinje cells, which induces cerebellar ataxia^59^. Surprisingly, we showed that *H. erinaceus* treatment potentially prevented the specific degeneration of Purkinje cells by enhancing ERK-CREB-PSD95 mechanisms in the 3-AP + 25 mg/kg H.E. group.

To further support the histological findings of neuroprotective effects of *H. erinaceus* on Purkinje cells in the cerebellum, our data showed a reduction of transcription factors for *calbindin-D28k* and *PCP4* genes in 3-AP + 10 mg/kg H.E. and 3-AP + 25 mg/kg H.E. animals compared to the 3-AP + saline group (Fig. 5G,H). The changes in transcript and protein levels of calbindin-D28k and PCP4 are important regulatory mechanisms for post-transcriptional processes of postmitotic neurons formation of Purkinje cells. Although no protein data was provided in the present study to support the notion of decrease in these transcription factors that used to produce the calbindin-D28k and PCP4 proteins, our results clearly suggest that the gain-of-function in *H. erinaceus* treated animals was mediated through ERK-CREB-PSD95 mechanisms to protect the Purkinje cells from degeneration induced by 3-AP. *PCP4* is highly specific for the Purkinje cell and it is a key modulator for calcium signalling in the developing brain of postmitotic neuroectoderm cells^69,70^. Of particular interest, overexpression of *PCP4* has been shown to affect the functional development of Purkinje cells with motor skill and learning impairment in the mouse models of Down syndrome during postnatal development^70,71^. Recently, it has been shown that the inhibition of calcium-mediated calpain activation is associated with the pro-survival effects of calbindin-D28K^72,73^. Although understanding of the interaction between calbindin-D28K and the pro-apoptotic protein caspase-3 remains obscure, our study has demonstrated a reduction of *caspase-3*, a crucial mediator of programmed cell death, in 3-AP + 25 mg/kg H.E. animals compared to the 3-AP + saline group. The anti-apoptotic effects of *H. erinaceus* could possibly be explained by the inhibition of capase-3 cleavage or calcium-mediated death signalling pathway, which protects against cell death from 3-AP.

To further investigate the effects of *H. erinaceus*, we conducted a correlation study on the relationships of the behavioural motor coordination and of the gene and protein functions. We found a significant positive correlation between the latency to fall and the percentage deficit by latency to fall in the 3-AP + 10 mg/kg and 25 mg/kg H.E. groups and the non-3-AP control group, but no correlation was found for the 3-AP + saline group, indicating *H. erinaceus* potentially rescued the behavioural motor deficits in 3-AP treated animals. Furthermore, we also observed a positive correlation in gene expression between the *CREB* and *TrkB*, as well as the correlation between PSD95 protein expression level and the percentage of latency to fall in the 3-AP + 25 mg/kg H.E. group, suggesting BDNF/TrkB/CREB and PSD95 have important roles in *H. erinaceus* rescue of the behavioural motor impairments in 3-AP-induced cerebellar ataxia.

Natural products have been used as traditional treatment without any documented adverse effects and many are still in use today. Culinary and medicinal mushrooms including *H. erinaceus* have been shown to have a wide range of bioactivities, including extending lifespan and delaying onset of age-related diseases^74^. To our knowledge, there are no reports of natural products that possess neuroprotective effects on acquired cerebellar ataxia, and this prompted us to examine the neuroprotective effects of *H. erinaceus* and to investigate its mechanisms in a neurodegenerative disorder model. Substantial evidence indicates that intrinsic free radical scavenging contributes to the neuroprotective effects of *H. erinaceus*^37^. Phenolic acids of *H. erinaceus* that possess hydroxyl groups have been shown to exert antioxidant properties by counteracting oxidative stress and other detrimental changes in brain tissues. Our findings that *H. erinaceus* has enhanced neuroplasticity functions were in agreement with previous studies on the neuritogenic and nerve regeneration effects of *H. erinaceus*^16,20,37,74–77^.

*Hericium erinaceus* has been reported to possess bioactive compounds (e.g. erinacines A-G) that can pass through the blood brain barrier and stimulate NGF synthesis^28–31^. Of particular interest, the standardised aqueous extract of *H. erinaceus* used in the present study contains 20.66% beta-glucan and 0.17% adenosine^37^. Beta-glucan derived from *Lentinus edodes* or Shiitake mushroom known as lentinan has been demonstrated to induce long-term potentiation in the rat dentate gyrus, indicating the potential effect of beta-glucan on neuroplasticity enhancement^78^. It has been shown that (1–3)-beta-glucan activates the influx of Ca^2+^ in the NR8383 macrophages through receptor-operated Ca^2+^ channels^79^. The influx of Ca^2+^ into the cell is through the NMDA receptors, and it eventually stimulates the neuronal nitric oxide synthase with the facilitation of PSD95, when other channels are less effective^80,81^. The induced nitric oxide activates the cGMP–protein kinase G (PKG) and ERK signalling, and ultimately, it leads to the increased expression of neuroplasticity-related proteins^82^. It has been shown that adenosine enhanced the production of nitric oxide^79^, and it reduced the neuronal damage by alteration of the lactate dehydrogenase release or deoxyglucose transport in primary cortical or hippocampal cell cultures that subjected to hypoxia or glucose deprivation^79,83^. Moreover, the adenosine receptors (A_1_ and A_2A_ receptors) were found to facilitate neuroprotective function by improving the excitotoxic neuronal damage in cell culture models of ischemia/hypoxia^83,84^. Interestingly, a similar neuroprotective effect of adenosine was also shown to attenuate neuronal cell death after the administration of potassium cyanide, an inhibitor of the mitochondrial respiratory chain, in another model of histotoxic anoxia^85^.

3-AP has been found to reduce the intracellular concentration of nicotinamide adenine dinucleotide (NAD^+^) and to interfere with zinc ion (Zn^2+^) homeostasis. This can be followed by impaired glyceraldehyde-3-phosphate dehydrogenase (GAPDH) activity, accumulation of dihydroxyacetone phosphate (DHAP), reduction in ATP production and increased expression of neuroplasticity and neurogenesis-related genes to reverse neuronal death. In this study, we found pERK1/2 protein expression was increased after treatment with 25 mg/kg *H. erinaceus*, and its neuroprotective effects against 3-AP-Iinduced neuronal cell death were mediated by Ras/Raf/MEK/ERK1/2 signalling pathway and by the regulation of pCREB (Fig. 6D). This observation was possibly due to the active compound of erinacines A-G from the *H. erinaceus* that enhanced the synthesis of NGF. Hypothetically, activation of tyrosine kinase receptors by NGF results in the recruitment of the cytoplasmic protein, Son of Sevenless (SOS), through intracellular Shc and Grb2 domains, which then activates the Ras/guanosine triphosphate complex^86^ and initiates cytoplasmic kinase signal transduction cascades^87^, and MEK1*/*2 catalyses the phosphorylation of ERK1*/*2 at Tyr204/187 mediated by importin-7, followed by translocation from the cytoplasm to the nucleus^88,89^. ERK1/2 acts as an upstream regulator by catalysing the phosphorylation of various cytoplasmic and nuclear substrates that encode transcription factors and gene regulatory proteins, including activators of transcription, c-Jun and c-Fos, as well as signal transducers, Elk1, CREB and c-Myc^87,90^. These transcription factors and gene regulatory proteins regulate the expression of proteins involved in survival, proliferation, and differentiation^87,88^. Thus, activation of Ras/Raf/MEK/ERK1/2 cascade protects and rescues Purkinje cells from 3-AP-induced neuronal death (Fig. 6D).

In conclusion, our present findings showed that 21-day intraperitoneal administration of *H. erinaceus* at the dosage of 25 mg/kg, but not 10 mg/kg, partially rescued behavioural motor deficit in rat model of cerebellar ataxia induced by 3-AP. Our results suggest that higher dose of *H. erinaceus* potentially rescues behavioural motor deficits through ERK-CREB-PSD95 neuroprotective mechanisms and prevent cerebellar Purkinje cell degeneration in rat model of 3-AP-induced cerebellar ataxia. Further in-depth investigations need to focus on the electrophysiological effects of *H. erinaceus* on spontaneous neuronal firing and intracellular calcium concentration in Purkinje cells of animal models of cerebellar ataxia.

## Supplementary information

Supplementary information

## References

[1] Klockgether T, Paulson H (2011). Milestones in ataxia. Mov Disord.

[2] Marsden JF, Day BL, Lord SR (2018). Chapter 17 - Cerebellar ataxia. Handbook of Clinical Neurology.

[3] Harding AE (1993). Clinical features and classification of inherited ataxias. Adv Neurol.

[4] Lim J (2006). A protein–protein interaction network for human inherited ataxias and disorders of Purkinje cell degeneration. Cell.

[5] Ferdinandusse S (2008). Ataxia with loss of Purkinje cells in a mouse model for Refsum disease. Proc. Natl. Acad. Sci..

[6] Sausbier M (2004). Cerebellar ataxia and Purkinje cell dysfunction caused by Ca2+-activated K+ channel deficiency. Proc. Natl. Acad. Sci..

[7] Marsden JF (2018). Cerebellar ataxia. Handb. Clin. Neurol..

[8] Keller JL, Bastian AJ (2014). A home balance exercise program improves walking in people with cerebellar ataxia. Neurorehabil Neural Repair.

[9] Rinninella E (2019). Food components and dietary habits: Keys for a healthy gut microbiota composition. Nutrients.

[10] Wang ZY, Fu HT (1981). Treatment of hereditary cerebellar ataxia with *Ganoderma capense.* Report of 4 cases. J. Tradit. Chin. Med..

[11] Thongbai B, Rapior S, Hyde KD, Wittstein K, Stadler M (2015). *Hericium erinaceus*, an amazing medicinal mushroom. Mycol. Prog..

[12] Khan M, Tania M, Liu R, Rahman M (2013). *Hericium erinaceus* : an edible mushroom with medicinal values. J. Complement. Integr. Med..

[13] Ratto D (2019). *Hericium erinaceus* improves recognition memory and induces hippocampal and cerebellar neurogenesis in frail mice during aging. Nutrients.

[14] Li IC (2018). Neurohealth properties of *Hericium erinaceus* mycelia enriched with erinacines. Behav Neurol.

[15] Vigna L (2019). *Hericium erinaceus* improves mood and sleep disorders in patients affected by overweight or obesity: could circulating pro-BDNF and BDNF be potential biomarkers?. Evid. Based Complement. Alternat. Med..

[16] Wong KH, Sabaratnam V, Abdullah N, Naidu M, Keynes R (2007). Activity of aqueous extracts of lion's mane mushroom *Hericium erinaceus* (Bull.: Fr.) Pers. (Aphyllophoromycetideae) on the neural cell line NG108–15. Int. J. Med.Mushrooms.

[17] Lai P-L (2013). Neurotrophic properties of *Hericium erinaceus* (Bull.: Fr.) Pers. grown in tropical climate of Malaysia.. Int. J. Med.Mushrooms.

[18] Phan C-W, David P, Naidu M, Wong K-H, Sabaratnam V (2013). Neurite outgrowth stimulatory effects of culinary-medicinal mushrooms and their toxicity assessment using differentiating Neuro-2a and embryonic fibroblast BALB/3T3. BMC Complement. Altern. Med..

[19] Wong K-H, Kanagasabapathy G, Bakar R, Phan C-W, Sabaratnam V (2017). Restoration of sensory dysfunction following peripheral nerve injury by the polysaccharide from culinary and medicinal mushroom, *Hericium erinaceus* (Bull.: Fr.) Pers. through its neuroregenerative action. Food Sci. Technol. (Campinas).

[20] Brandalise F (2017). Dietary supplementation of *Hericium erinaceus* increases mossy Fiber-CA3 hippocampal neurotransmission and recognition memory in wild-type mice. Evid. Based Complement. Alternat. Med..

[21] Mori K, Inatomi S, Ouchi K, Azumi Y, Tuchida T (2009). Improving effects of the mushroom Yamabushitake (*Hericium erinaceus*) on mild cognitive impairment: a double-blind placebo-controlled clinical trial. Phytother. Res..

[22] Inanaga K (2014). Marked improvement of neurocognitive impairment after treatment with compounds from Hericium erinaceum: a case study of recurrent depressive disorder. Pers. Med. Univers..

[23] Ryu S, Kim HG, Kim JY, Kim SY, Cho KO (2018). *Hericium erinaceus* extract reduces anxiety and depressive behaviors by promoting hippocampal neurogenesis in the adult mouse brain. J. Med. Food.

[24] Nagano M (2010). Reduction of depression and anxiety by 4 weeks *Hericium erinaceus* intake. Biomed Res.

[25] Chong PS, Fung ML, Wong KH, Lim LW (2020). Therapeutic potential of *Hericium erinaceus* for depressive disorder. Int. J. Mol. Sci..

[26] Limanaqi F (2020). Potential antidepressant effects of *Scutellaria baicalensis*, *Hericium erinaceus* and *Rhodiola rosea*. Antioxidants (Basel).

[27] Chiu CH (2018). Erinacine A-enriched *Hericium erinaceus* mycelium produces antidepressant-like effects through modulating BDNF/PI3K/Akt/GSK-3beta signaling in mice. Int. J. Mol. Sci..

[28] Kawagishi H (1991). Hericenones C, D and E, stimulators of nerve growth factor (NGF)-synthesis, from the mushroom *Hericium erinaceum*. Tetrahedron Lett..

[29] Kawagishi H (1996). Erinacines E, F, and G, stimulators of nerve growth factor (NGF)-synthesis, from the mycelia of *Hericium erinaceum*. Tetrahedron Lett..

[30] Kawagishi H (1994). Erinacines A, B and C, strong stimulators of nerve growth factor (NGF)-synthesis, from the mycelia of *Hericium erinaceum*. Tetrahedron Lett..

[31] Kawagishi H (1996). Erinacine D, a stimulator of NGF-synthesis, from the mycelia of *Hericium erinaceum*. Heterocycl. Commun..

[32] Hwang J-H (2013). *Hericium erinaceus* enhances neurotrophic factors and prevents cochlear cell apoptosis in senescence accelerated mice. J. Funct. Foods.

[33] Mori K (2008). Nerve growth factor-inducing activity of *Hericium erinaceus* in 1321N1 human astrocytoma cells. Biol. Pharm. Bull..

[34] Habtemariam S (2018). The brain-derived neurotrophic factor in neuronal plasticity and neuroregeneration: new pharmacological concepts for old and new drugs. Neural Regen. Res..

[35] Skaper SD (2008). The biology of neurotrophins, signalling pathways, and functional peptide mimetics of neurotrophins and their receptors. CNS Neurol Disord Drug Targets.

[36] Lakshmanan H (2016). Haematological, biochemical and histopathological aspects of *Hericium erinaceus* ingestion in a rodent model: A sub-chronic toxicological assessment. J. Ethnopharmacol..

[37] Lew S-Y, Yow Y-Y, Lim L-W, Wong K-H (2019). Antioxidant-mediated protective role of Hericium erinaceus (Bull.: Fr.) Pers. against oxidative damage in fibroblasts from Friedreich’s ataxia patient. Food Sci. Technol..

[38] Abada YS, Nguyen HP, Schreiber R, Ellenbroek B (2013). Assessment of motor function, sensory motor gating and recognition memory in a novel BACHD transgenic rat model for huntington disease. PLoS ONE.

[39] Fischer AH, Jacobson KA, Rose J, Zeller R (2008). Hematoxylin and eosin staining of tissue and cell sections. Cold Spring Harb. Protoc..

[40] Moers-Hornikx VM (2011). Periaqueductal grey stimulation induced panic-like behaviour is accompanied by deactivation of the deep cerebellar nuclei. Cerebellum.

[41] Louis ED, Babij R, Lee M, Cortés E, Vonsattel J-PG (2013). Quantification of cerebellar hemispheric purkinje cell linear density: 32 ET cases versus 16 controls. Mov. Disord..

[42] Lim LW (2016). Tetratricopeptide repeat domain 9A modulates anxiety-like behavior in female mice. Sci Rep.

[43] Livak KJ, Schmittgen TD (2001). Analysis of relative gene expression data using real-time quantitative PCR and the 2(-Delta Delta C(T)) Method. Methods.

[44] Xu H (2016). Effects of Duloxetine Treatment on Cognitive Flexibility and BDNF Expression in the mPFC of Adult Male Mice Exposed to Social Stress during Adolescence. Front Mol Neurosci.

[45] Wagner N (2005). Coronary vessel development requires activation of the TrkB neurotrophin receptor by the Wilms' tumor transcription factor Wt1. Genes Dev.

[46] Wang HY (2015). RBFOX3/NeuN is Required for Hippocampal Circuit Balance and Function. Sci Rep.

[47] Kerr B, Silva PA, Walz K, Young JI (2010). Unconventional transcriptional response to environmental enrichment in a mouse model of Rett syndrome. PLoS ONE.

[48] Konirova J (2017). Modulated DISP3/PTCHD2 expression influences neural stem cell fate decisions. Sci. Rep..

[49] Wong YW (2007). Gene expression analysis of nuclear factor I-A deficient mice indicates delayed brain maturation. Genome Biol..

[50] Cai W, Zhang Z, Huang Y, Sun H, Qiu L (2018). Vaccarin alleviates hypertension and nephropathy in renovascular hypertensive rats. Exp. Ther. Med..

[51] Xiao J (2008). Expression of Pcp4 gene during osteogenic differentiation of bone marrow mesenchymal stem cells in vitro. Mol. Cell Biochem..

[52] Bolaños A (2012). Regulation of calbindin-D(28k) expression by Msx2 in the dental epithelium. J. Histochem. Cytochem..

[53] Bhaskar Y, Lim LW, Mitra R (2018). Enriched environment facilitates anxiolytic efficacy driven by deep-brain stimulation of medial prefrontal cortex. Front. Behav. Neurosci..

[54] Liu A, Jain N, Vyas A, Lim LW (2015). Ventromedial prefrontal cortex stimulation enhances memory and hippocampal neurogenesis in the middle-aged rats. eLife..

[55] Lim LW (2015). Electrical stimulation alleviates depressive-like behaviors of rats: investigation of brain targets and potential mechanisms. Transl. Psychiatry.

[56] Costenla AR, Cunha RA, de Mendonca A (2010). Caffeine, adenosine receptors, and synaptic plasticity. J. Alzheimers Dis..

[57] Alp H (2012). Protective effects of beta glucan and gliclazide on brain tissue and sciatic nerve of diabetic rats induced by streptozosin. J. Diabetes Res..

[58] Jiang YY, Cao BB, Wang XQ, Peng YP, Qiu YH (2015). Cerebellar ataxia induced by 3-AP affects immunological function. Neuroendocrinol. Lett.

[59] Kaffashian M (2011). Profound alterations in the intrinsic excitability of cerebellar Purkinje neurons following neurotoxin 3-acetylpyridine (3-AP)-induced ataxia in rat: new insights into the role of small conductance K+ channels. Physiol. Res..

[60] Sukin D (1987). Temporal sequence of motor disturbances and increased cerebellar glutamic acid decarboxylase activity following 3-acetylpyridine lesions in adult rats. Brain Res..

[61] Decimo I (2011). Nestin- and doublecortin-positive cells reside in adult spinal cord meninges and participate in injury-induced parenchymal reaction. Stem Cells (Dayton, Ohio).

[62] Koh S-H, Park H-H (2017). Neurogenesis in stroke recovery. Transl. Stroke Res..

[63] Jin K (2006). Evidence for stroke-induced neurogenesis in the human brain. Proc. Natl. Acad. Sci..

[64] Macas J, Nern C, Plate KH, Momma S (2006). Increased generation of neuronal progenitors after ischemic injury in the aged adult human forebrain. J. Neurosci..

[65] Encinas JM (2011). Division-coupled astrocytic differentiation and age-related depletion of neural stem cells in the adult hippocampus. Cell Stem Cell.

[66] Butterworth RF, Hamel E, Landreville F, Barbeau A (1978). Cerebellar ataxia produced by 3-acetyl pyridine in rat. Can. J. Neurol. Sci..

[67] Karachot L, Ito M, Kanai Y (1987). Long-term effects of 3-acetylpyridine-induced destruction of cerebellar climbing fibers on Purkinje cell inhibition of vestibulospinal tract cells of the rat. Exp. Brain Res..

[68] Mohammadi R, Heidari MH, Sadeghi Y, Abdollahifar MA, Aghaei A (2018). Evaluation of the spatial arrangement of Purkinje cells in ataxic rat's cerebellum after Sertoli cells transplantation. Folia Morphol. (Warsz).

[69] Bulfone A (2004). Pcp4l1, a novel gene encoding a Pcp4-like polypeptide, is expressed in specific domains of the developing brain. Gene Expr. Patterns.

[70] Mouton-Liger F (2014). Developmental molecular and functional cerebellar alterations induced by PCP4/PEP19 overexpression: implications for Down syndrome. Neurobiol. Dis..

[71] Laffaire J (2009). Gene expression signature of cerebellar hypoplasia in a mouse model of Down syndrome during postnatal development. BMC Genomics.

[72] Choi WS, Lee E, Lim J, Oh YJ (2008). Calbindin-D28K prevents drug-induced dopaminergic neuronal death by inhibiting caspase and calpain activity. Biochem. Biophys. Res. Commun..

[73] Porter AG, Janicke RU (1999). Emerging roles of caspase-3 in apoptosis. Cell Death Differ..

[74] Phan CW, David P, Naidu M, Wong KH, Sabaratnam V (2015). Therapeutic potential of culinary-medicinal mushrooms for the management of neurodegenerative diseases: diversity, metabolite, and mechanism. Crit. Rev. Biotechnol..

[75] Phan CW (2014). Hericium erinaceus (Bull.: Fr) Pers. cultivated under tropical conditions: isolation of hericenones and demonstration of NGF-mediated neurite outgrowth in PC12 cells via MEK/ERK and PI3K-Akt signaling pathways. Food Funct..

[76] Wong KH (2011). Peripheral nerve regeneration following crush injury to rat peroneal nerve by aqueous extract of medicinal mushroom *Hericium erinaceus* (Bull.: Fr) Pers. (Aphyllophoromycetideae). Evid. Based Complement. Alternat. Med.

[77] Wong KH, Ng CC, Kanagasabapathy G, Yow YY, Sabaratnam V (2017). An overview of culinary and medicinal mushrooms in neurodegeneration and neurotrauma research. Int. J. Med. Mushrooms.

[78] Edagawa Y, Smriga M, Nishiyama N, Saito H (2001). Systemic administration of lentinan, a branched beta-glucan, enhances long-term potentiation in the rat dentate gyrus in vivo. Neurosci. Lett..

[79] Mork AC (1998). Effects of particulate and soluble (1–3)-beta-glucans on Ca2+ influx in NR8383 alveolar macrophages. Immunopharmacology.

[80] Garthwaite J, Charles SL, Chess-Williams R (1988). Endothelium-derived relaxing factor release on activation of NMDA receptors suggests role as intercellular messenger in the brain. Nature.

[81] Kiedrowski L, Costa E, Wroblewski JT (1992). Glutamate receptor agonists stimulate nitric oxide synthase in primary cultures of cerebellar granule cells. J. Neurochem..

[82] Gallo EF, Iadecola C (2011). Neuronal nitric oxide contributes to neuroplasticity-associated protein expression through cGMP, protein kinase G, and extracellular signal-regulated kinase. J. Neurosci..

[83] Goldberg MP, Monyer H, Weiss JH, Choi DW (1988). Adenosine reduces cortical neuronal injury induced by oxygen or glucose deprivation in vitro. Neurosci. Lett..

[84] de Mendonça A, Sebastião AM, Ribeiro JA (2000). Adenosine: does it have a neuroprotective role after all?. Brain Res. Rev..

[85] Christopher DS, William AF, Kong-Woo Y (1993). Attenuation of potassium cyanide-mediated neuronal cell death by adenosine. J. Neurosurg..

[86] Gureasko J (2008). Membrane-dependent signal integration by the Ras activator Son of sevenless. Nat. Struct. Mol. Biol..

[87] Zou J (2019). Mechanisms shaping the role of ERK1/2 in cellular senescence (Review). Mol. Med. Rep..

[88] Chuderland D, Konson A, Seger R (2008). Identification and characterization of a general nuclear translocation signal in signaling proteins. Mol. Cell.

[89] Matsubayashi Y, Fukuda M, Nishida E (2001). Evidence for existence of a nuclear pore complex-mediated, cytosol-independent pathway of nuclear translocation of ERK MAP kinase in permeabilized cells. J. Biol. Chem..

[90] Roskoski R (2012). ERK1/2 MAP kinases: structure, function, and regulation. Pharmacol. Res..

