# Supplementary material for: Hericium erinaceus potentially rescues behavioural motor deficits through ERK-CREB-PSD95 neuroprotective mechanisms in rat model of 3-acetylpyridine-induced cerebellar ataxia
Source: Sci Rep. 2020 Sep 10;10:14945. doi: 10.1038/s41598-020-71966-z (PMC7483741; doi:10.1038/s41598-020-71966-z)
Supplement: Supplementary file 1 — Supplementary information [file 41598_2020_71966_MOESM1_ESM.pdf]

***Hericium erinaceus* potentially rescues behavioural motor deficits through ERK-CREB-PSD95 neuroprotective mechanisms in rat model of 3-acetylpyridine-induced cerebellar ataxia**

[1 #]Pit Shan CHONG, [1 #]Sharafuddin KHAIRUDDIN, [1]Anna Chung Kwan TSE, [1]Lih Fhung HIEW, [1]Chun Lok LAU, [1]George Lim TIPOE, [1]Man-Lung FUNG, [1,2]Kah Hui WONG, [1]Lee Wei LIM

Supplementary information

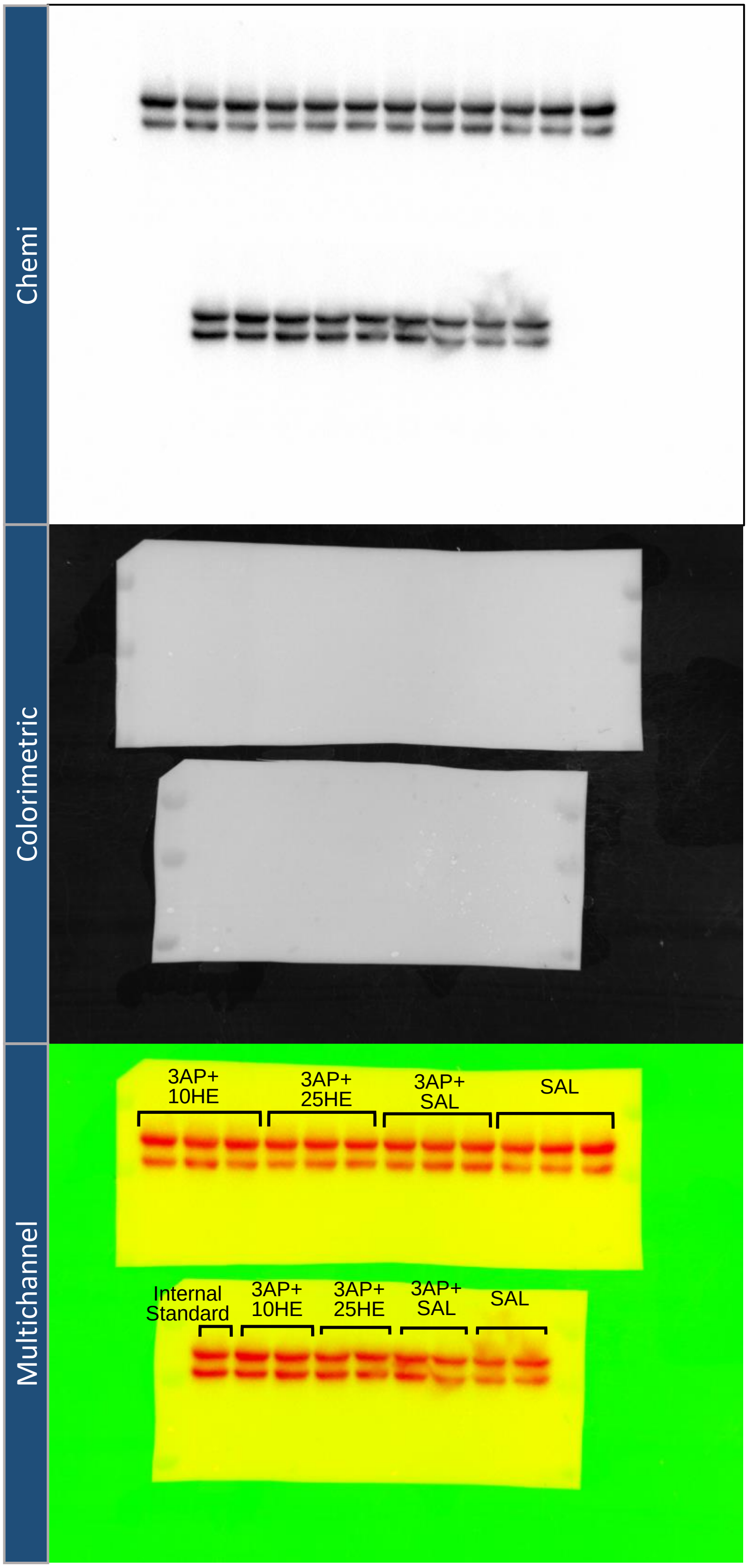

Figure S1: Western blot analysis of ERK1/2. Two blots were exposed and captured in one image. The lower blot is used as a representative figure in the manuscript without including the internal standard.

Hericium erinaceus **potentially rescues behavioural motor deficits through ERK-CREB-PSD95 neuroprotective mechanisms in rat model of 3-acetylpyridine-induced cerebellar ataxia**

[1 #]Pit Shan CHONG, [1 #]Sharafuddin KHAIRUDDIN, [1]Anna Chung Kwan TSE, [1]Lih Fhung HIEW, [1]Chun Lok LAU, [1]George Lim TIPOE, [1]Man-Lung FUNG, [1,2]Kah Hui WONG, [1]Lee Wei LIM

Supplementary information

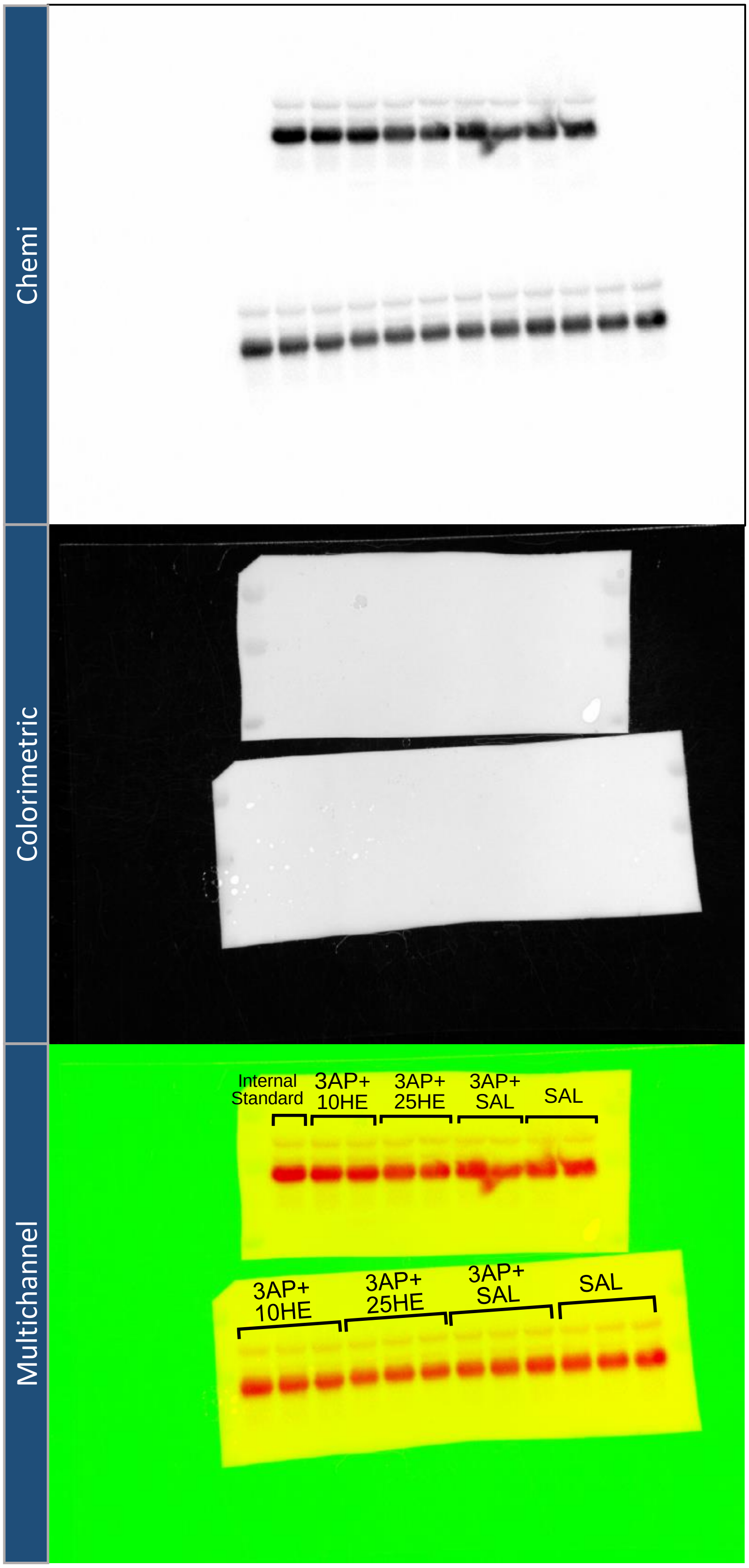

Figure S2: Western blot of GAPDH as a loading control for ERK1/2. The upper blot is used as a representative figure in the manuscript without including the internal standard.

[1 #]Pit Shan CHONG, [1 #]Sharafuddin KHAIRUDDIN, [1]Anna Chung Kwan TSE, [1]Lih Fhung HIEW, [1]Chun Lok LAU, [1]George Lim TIPOE, [1]Man-Lung FUNG, [1,2]Kah Hui WONG, [1]Lee Wei LIM

Supplementary information

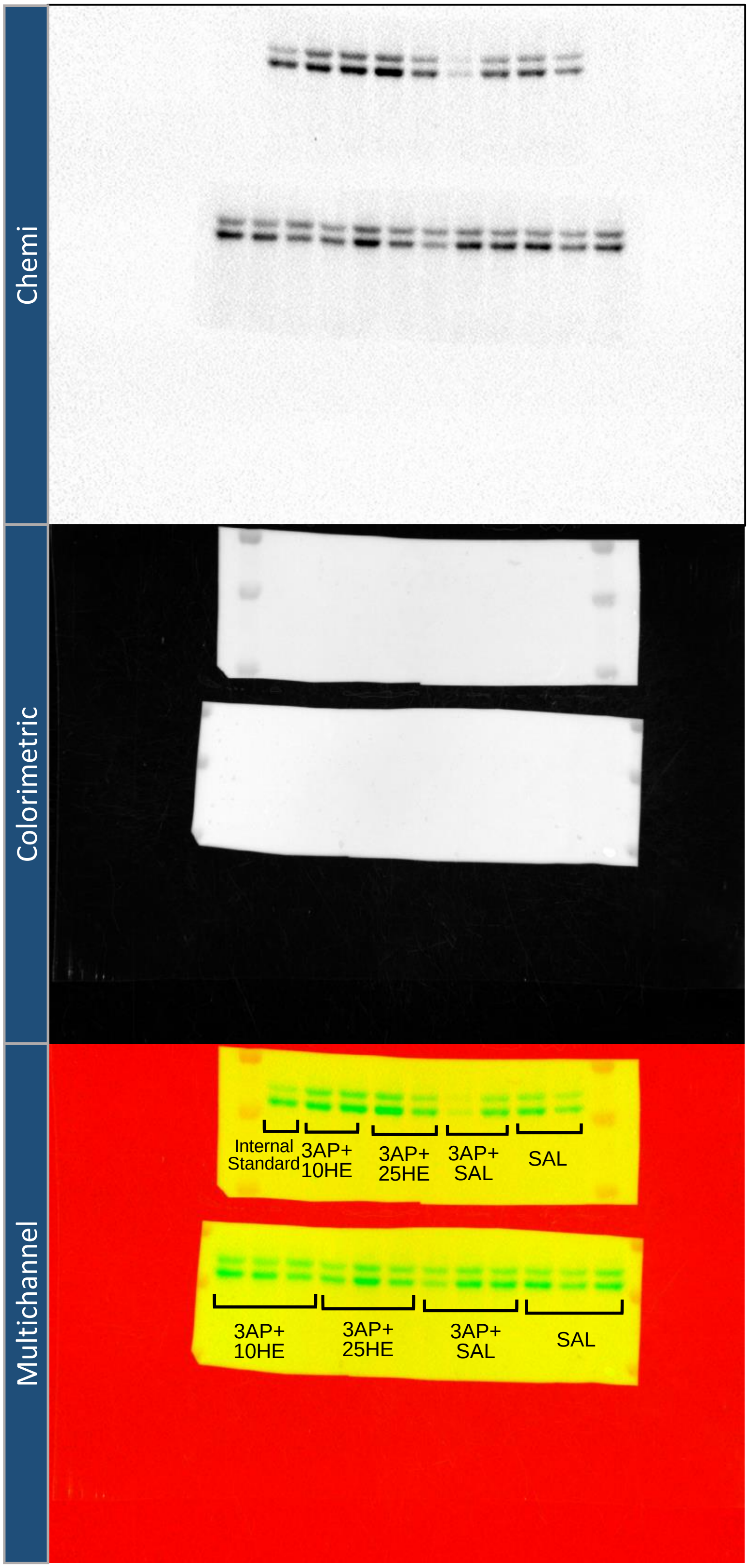

Figure S3: Western blot analysis of pERK1/2. Two blots were exposed and captured in one image. The upper blot is used as a representative figure in the manuscript without including the internal standard.

Hericium erinaceus potentially rescues behavioural motor deficits through ERK-CREB-PSD95 neuroprotective mechanisms in rat model of 3-acetylpyridine-induced cerebellar ataxia

[1 #]Pit Shan CHONG, [1 #] Sharafuddin KHAIRUDDIN, [1] Anna Chung Kwan TSE, [1] Lih Fhung HIEW, [1] Chun Lok LAU, [1] George Lim TIPOE, [1] Man-Lung FUNG, [1,2] Kah Hui WONG, [1] Lee Wei LIM

Supplementary information

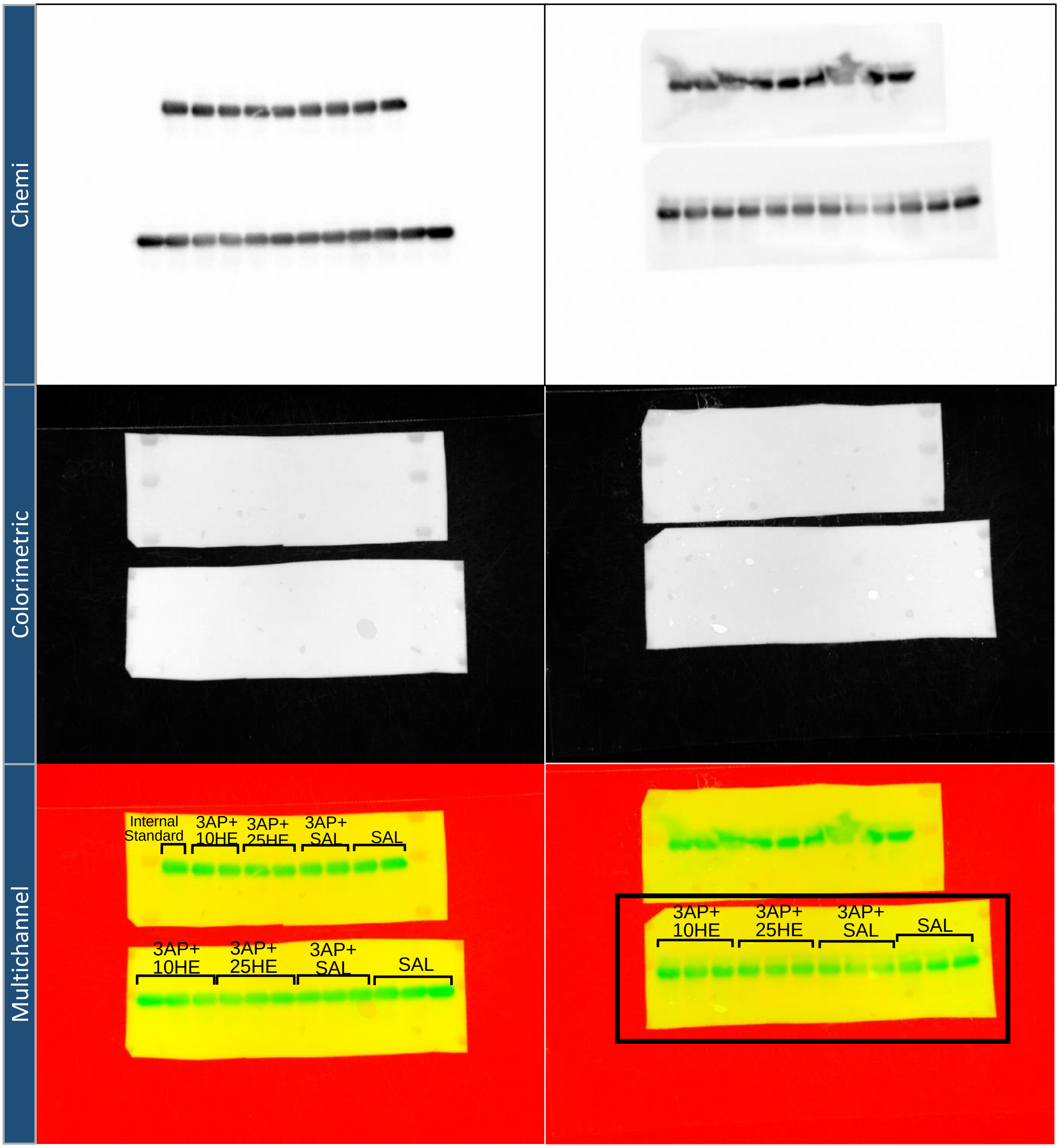

Figure S4 : Western blot of GAPDH as a loading control for pERK1/2, SYP (Gel 1) and PSD-95 (left). Two blots were exposed and captured in one image. The upper blot is used as a representative figure in the manuscript without including the internal standard. **Western Blot of GAPDH as a loading control for SYP (Gel 2) (right).**

Hericium erinaceus potentially rescues behavioural motor deficits through ERK-CREB-PSD95 neuroprotective mechanisms in rat model of 3-acetylpyridine-induced cerebellar ataxia

[1#]Pit Shan CHONG, [1#]Sharafuddin KHAIRUDDIN, [1]Anna Chung Kwan TSE, [1]Lih Fhung HIEW, [1]Chun Lok LAU, [1]George Lim TIPOE, [1]Man-Lung FUNG, [1,2]Kah Hui WONG, [1]Lee Wei LIM

Supplementary information

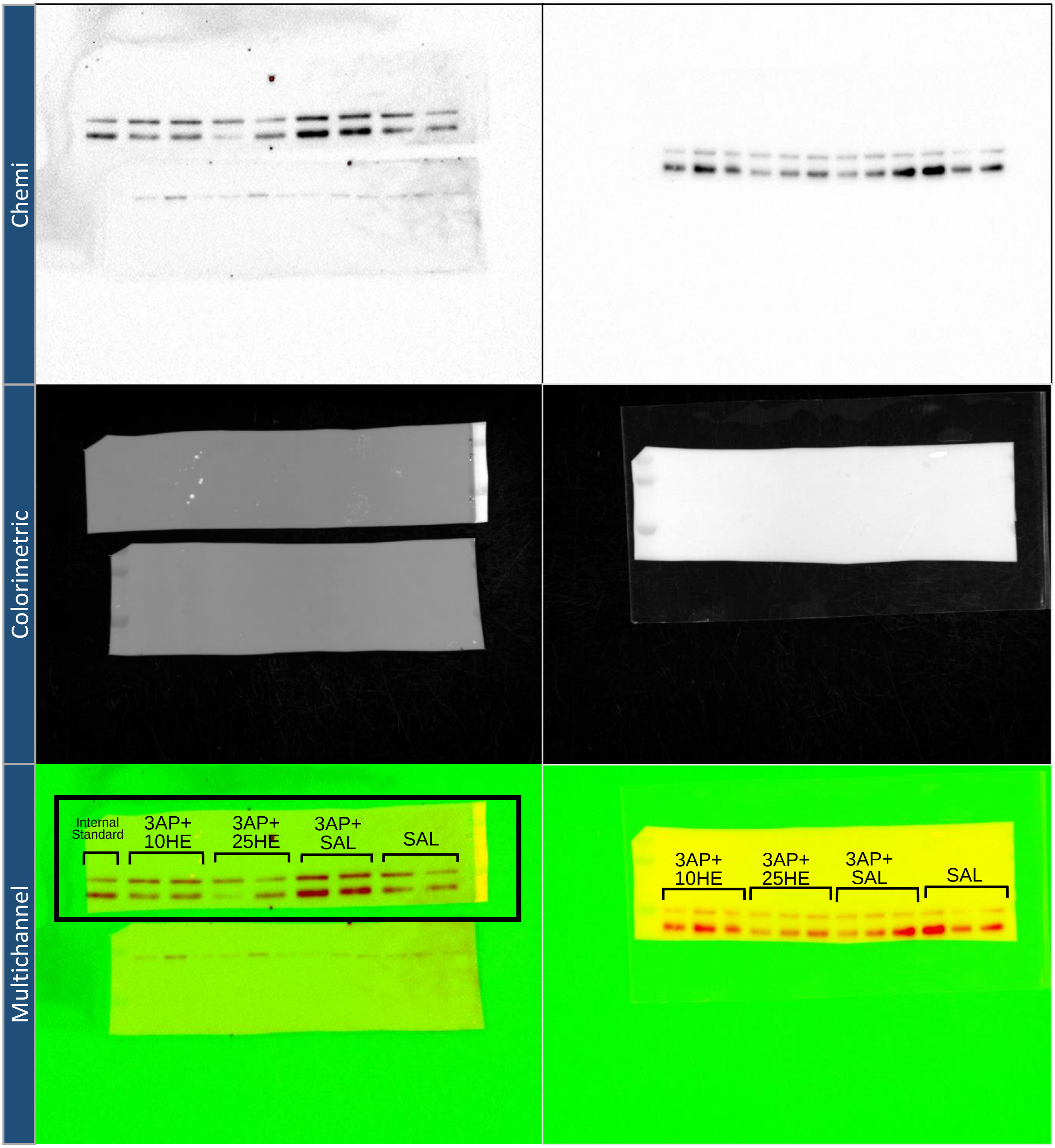

Figure S5: Western blot analysis of CREB. The upper left blot is used as a representative figure in the manuscript without including the internal standard.

Hericium erinaceus **potentially rescues behavioural motor deficits through ERK-CREB-PSD95 neuroprotective mechanisms in rat model of 3-acetylpyridine-induced cerebellar ataxia**

[1 #]Pit Shan CHONG, [1 #] Sharafuddin KHAIRUDDIN, [1] Anna Chung Kwan TSE, [1] Lih Fhung HIEW, [1] Chun Lok LAU, [1] George Lim TIPOE, [1] Man-Lung FUNG, [1,2] Kah Hui WONG, [1] Lee Wei LIM

Supplementary information

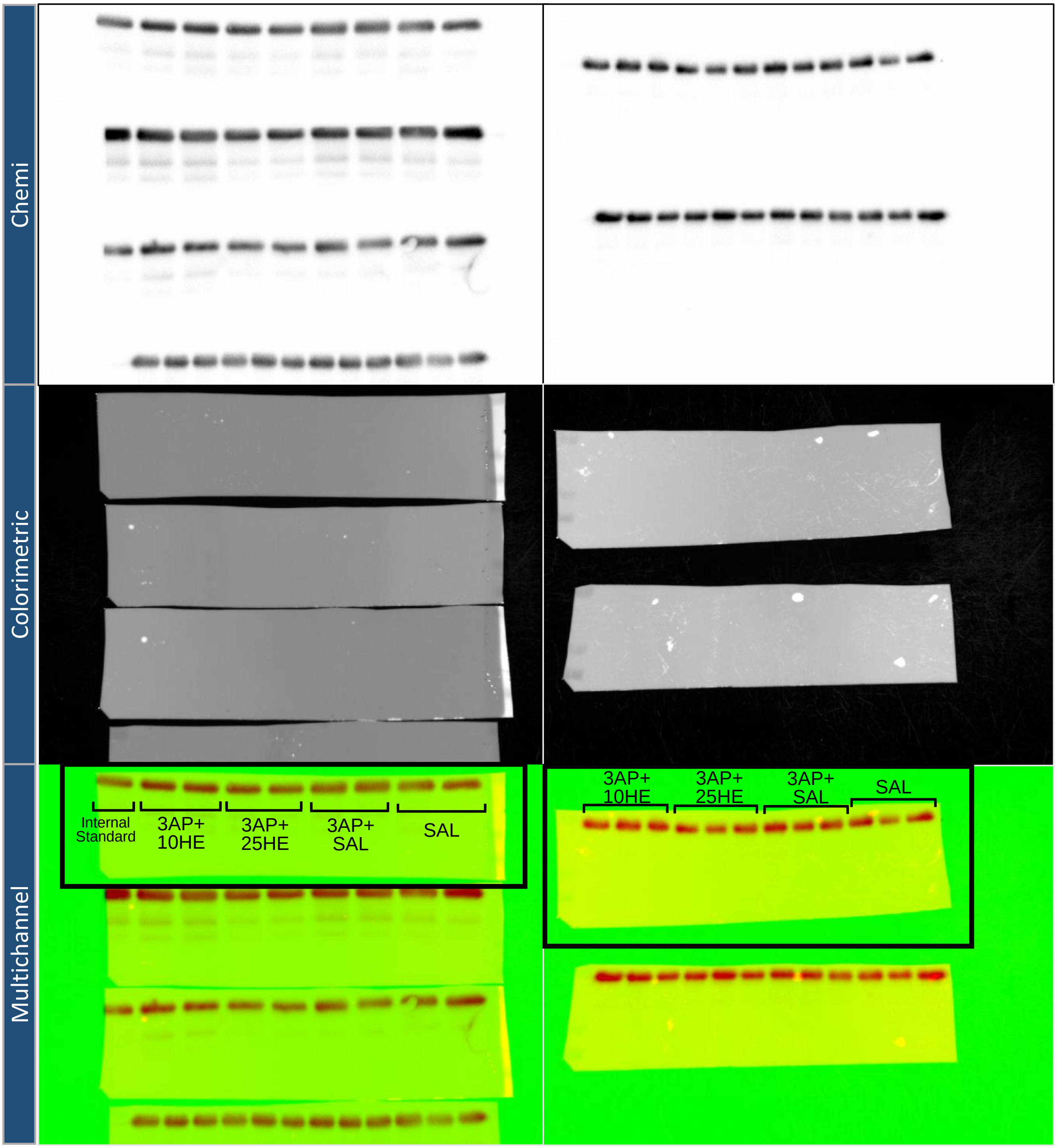

Figure S6 : Western blot of GAPDH as a loading control for CREB. More than one membrane were exposed at the same time while the blots of GAPDH were labelled. The top left blot is used as a representative figure in the manuscript without including the internal standard.

Hericium erinaceus **potentially rescues behavioural motor deficits through ERK-CREB-PSD95 neuroprotective mechanisms in rat model of 3-acetylpyridine-induced cerebellar ataxia**

[1 #]Pit Shan CHONG, [1 #] Sharafuddin KHAIRUDDIN, [1] Anna Chung Kwan TSE, [1] Lih Fhung HIEW, [1] Chun Lok LAU, [1] George Lim TIPOE, [1] Man-Lung FUNG, [1,2] Kah Hui WONG, [1] Lee Wei LIM

Supplementary information

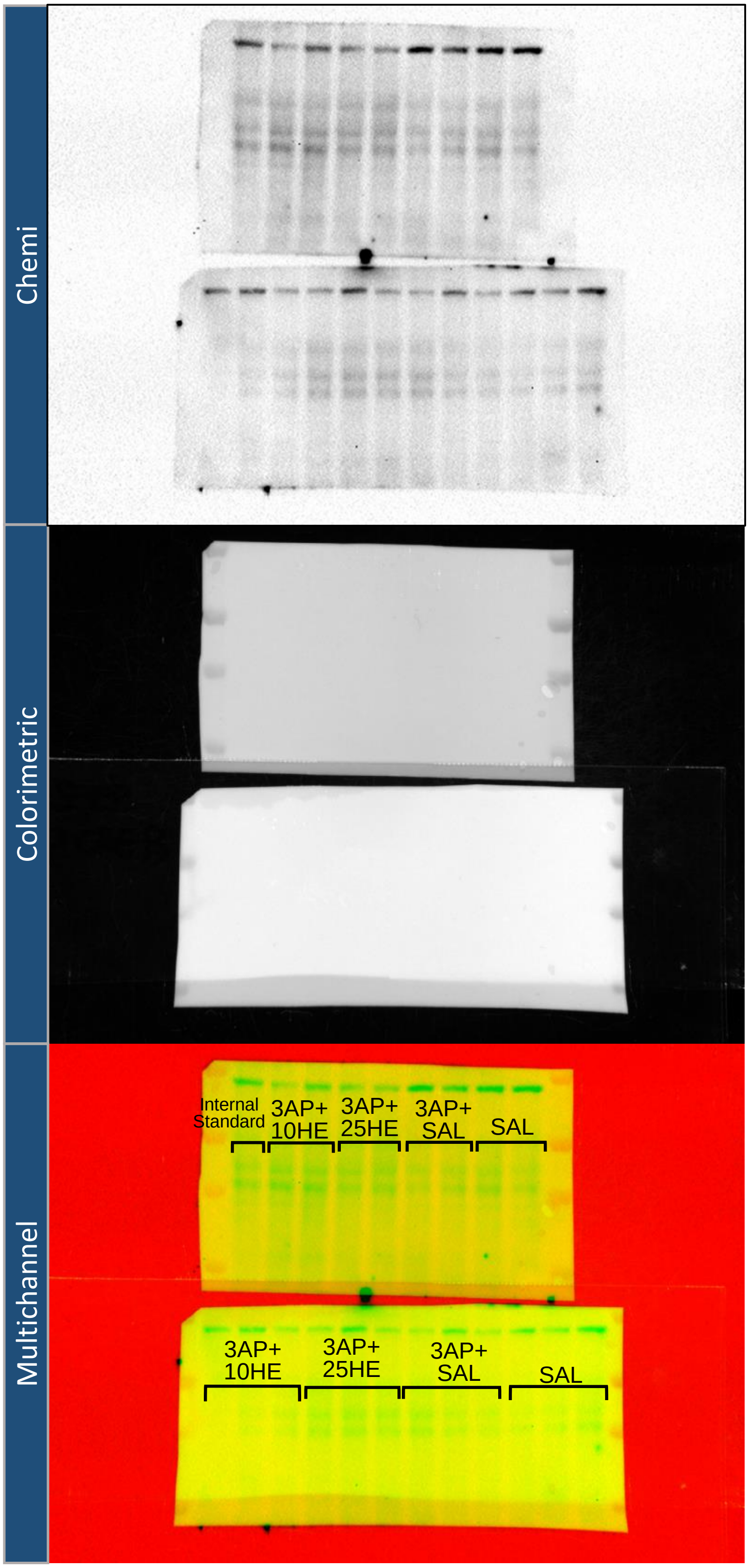

Figure S7: Western blot analysis of pCREB. Two blots were exposed and captured in one image. The upper blot is used as a representative figure in the manuscript without including the internal standard.

Hericium erinaceus potentially rescues behavioural motor deficits through ERK-CREB-PSD95 neuroprotective mechanisms in rat model of 3-acetylpyridine-induced cerebellar ataxia

[1 #]Pit Shan CHONG, [1 #]Sharafuddin KHAIRUDDIN, [1]Anna Chung Kwan TSE, [1]Lih Fhung HIEW, [1]Chun Lok LAU, [1]George Lim TIPOE, [1]Man-Lung FUNG, [1,2]Kah Hui WONG, [1]Lee Wei LIM

Supplementary information

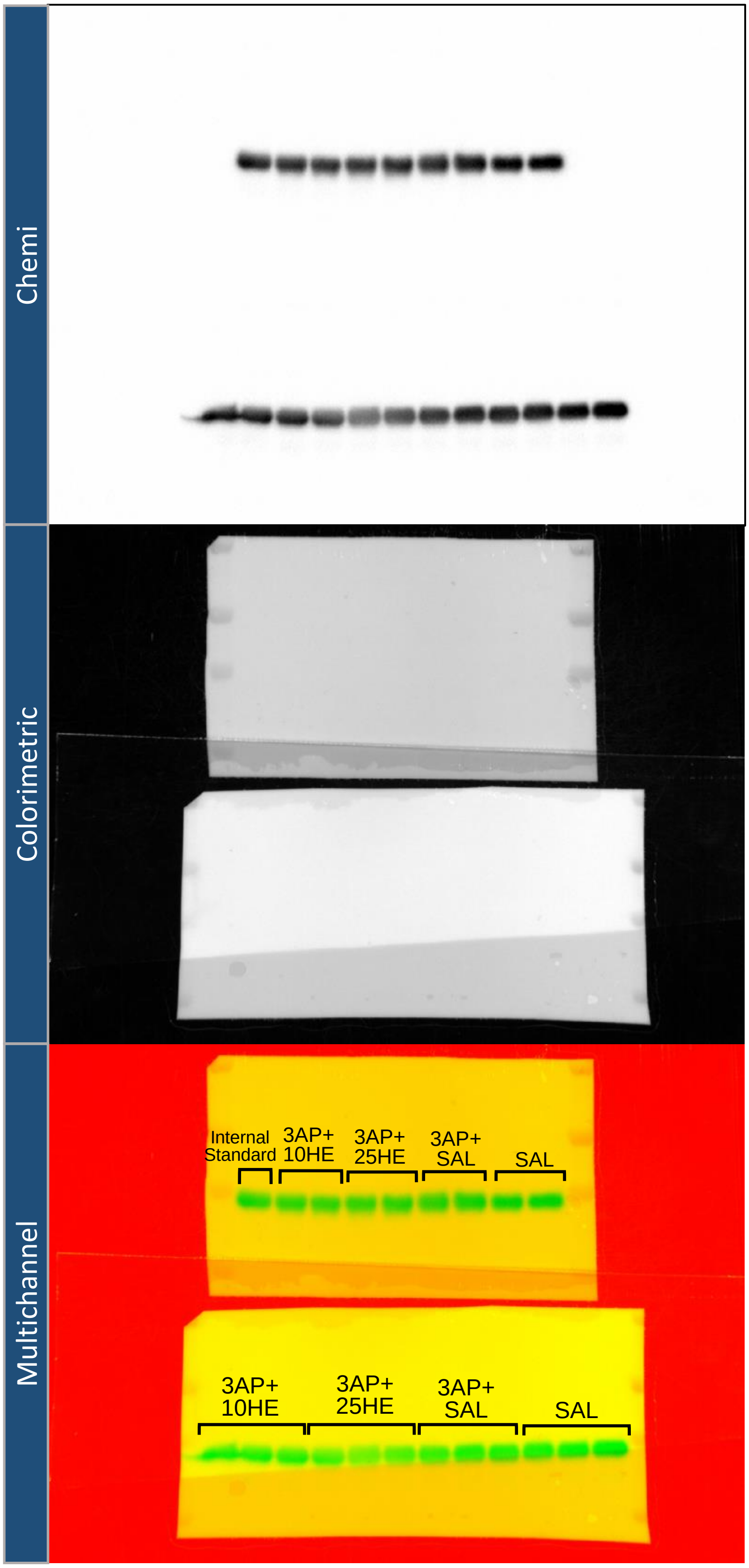

Figure S8 : Western blot of GAPDH as a loading control for pCREB. . Two blots were exposed and captured in one image. The upper blot is used as a representative figure in the manuscript without including the internal standard.

***Hericium erinaceus* potentially rescues behavioural motor deficits through ERK-CREB-PSD95 neuroprotective mechanisms in rat model of 3-acetylpyridine-induced cerebellar ataxia**

[1#]Pit Shan CHONG, [1#]Sharafuddin KHAIRUDDIN, [1]Anna Chung Kwan TSE, [1]Lih Fhung HIEW, [1]Chun Lok LAU, [1]George Lim TIPOE, [1]Man-Lung FUNG, [1,2]Kah Hui WONG, [1]Lee Wei LIM

Supplementary information

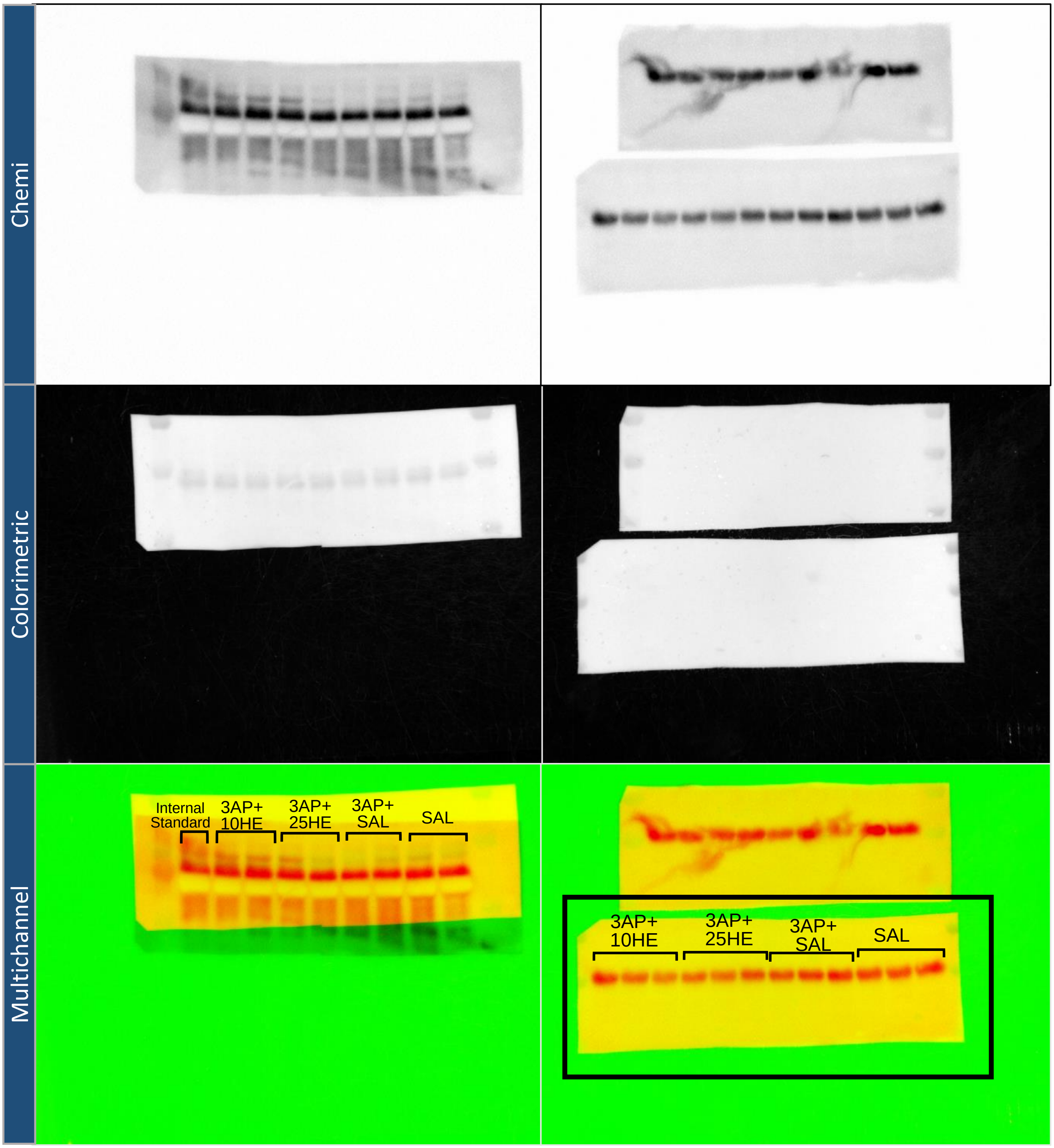

Figure S9: Western blot analysis of SYP. The left blot is used as a representative figure in the manuscript without including the internal standard. The left membrane was moved while imaging for colorimetric image, thus the image of multichannel was not merge perfectly.

[1 #]Pit Shan CHONG, [1 #]Sharafuddin KHAIRUDDIN, [1]Anna Chung Kwan TSE, [1]Lih Fhung HIEW, [1]Chun Lok LAU, [1]George Lim TIPOE, [1]Man-Lung FUNG, [1,2]Kah Hui WONG, [1]Lee Wei LIM

Supplementary information

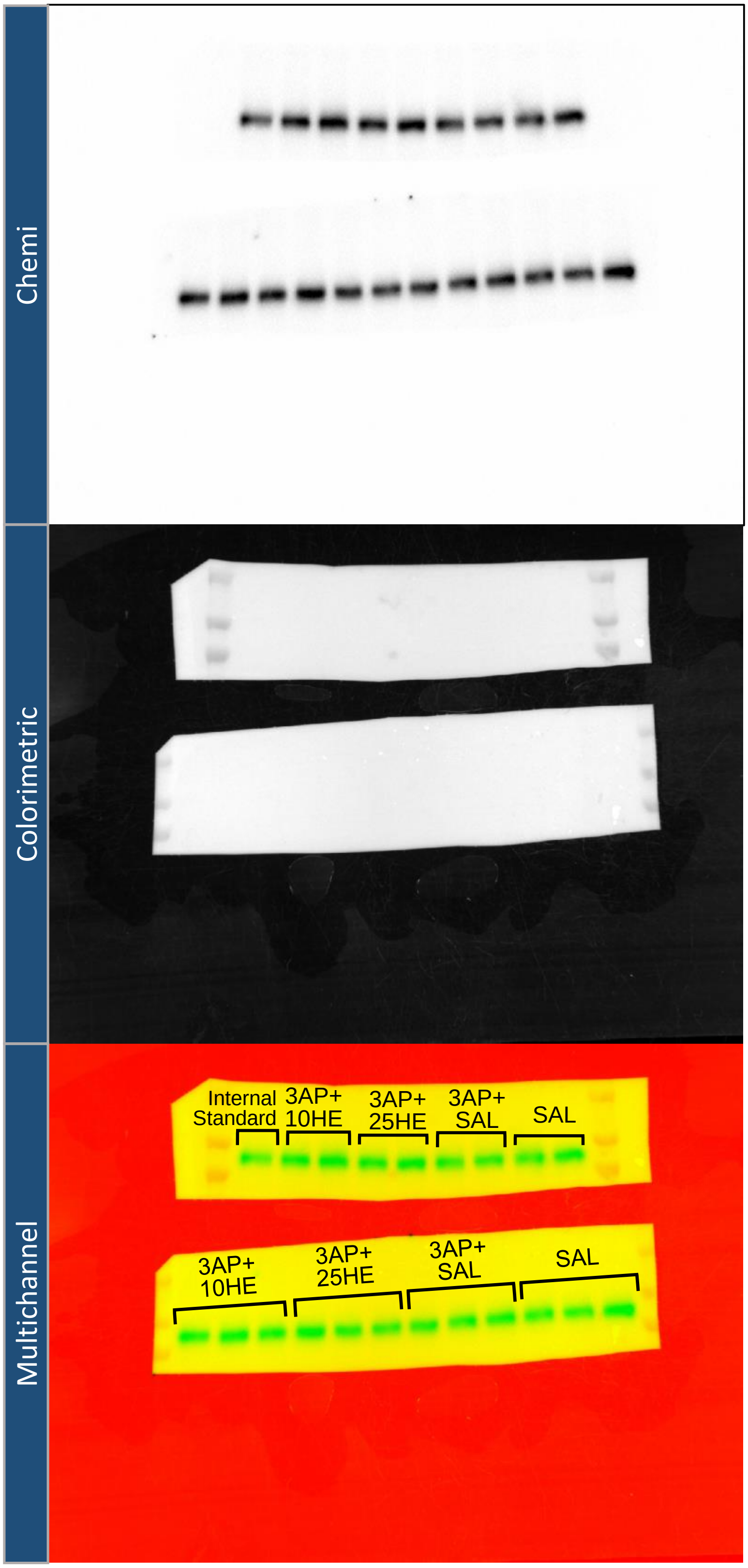

Figure S10: Western blot analysis of PSD-95. Four blots were exposed and captured in two image and blots for PSD-95 were labelled. The upper blot is used as a representative figure in the manuscript without including the internal standard.

Hericium erinaceus **potentially rescues behavioural motor deficits through ERK-CREB-PSD95 neuroprotective mechanisms in rat model of 3-acetylpyridine-induced cerebellar ataxia**

[1 #]Pit Shan CHONG, [1 #] Sharafuddin KHAIRUDDIN, [1] Anna Chung Kwan TSE, [1] Lih Fhung HIEW, [1] Chun Lok LAU, [1] George Lim TIPOE, [1] Man-Lung FUNG, [1,2] Kah Hui WONG, [1] Lee Wei LIM

Supplementary information

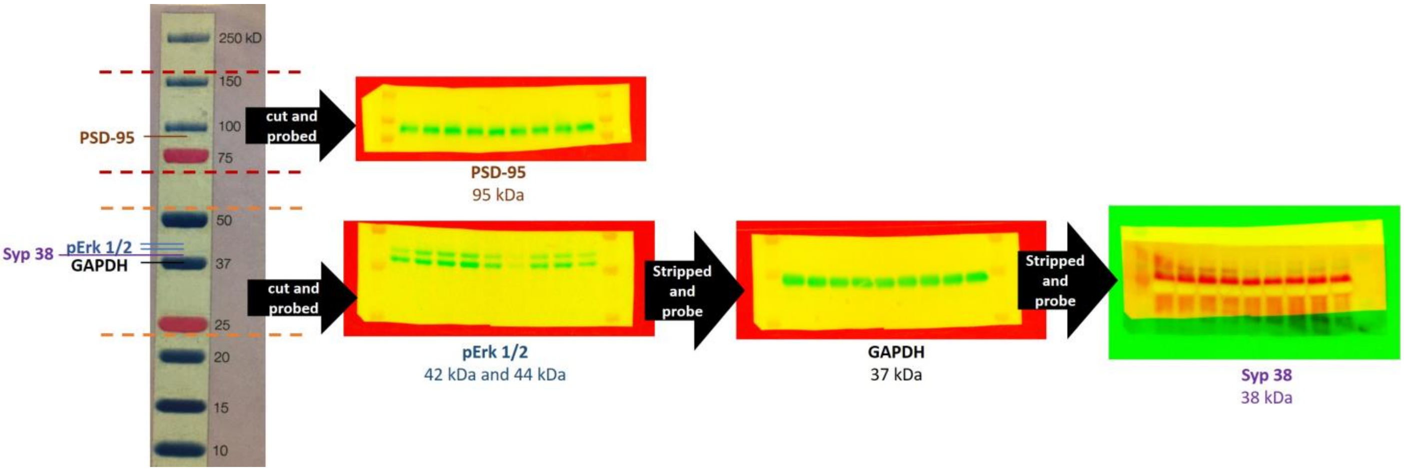

Figure S11: **Schematic diagram of the probing process of blot for PSD95, pERK1/2, SYP (Gel 1) and GAPDH**. PSD95, pERK1/2, SYP (Gel 1) and GAPDH were probed from the same blot with the same samples. The membrane was cut for detecting PSD95 and pERK1/2 separately. pERK1/2 were then stripped and probed with GAPDH. SYP (Gel 1) were further probed after stripping the GAPDH.
